# Ferroptosis in smoke inhalation injury: from mechanisms to potential therapeutic targets

**DOI:** 10.3389/fcell.2026.1832061

**Published:** 2026-09-15

**Authors:** Nayab Ibrahim, Jinchao Zhu, Yanqing Liu, Chuanlong Li, Ningning Shao, Wenping Fan, Yue Shi, Jianxin Li, Jinrui Dong, Haojun Fan

**Affiliations:** 1 Key Laboratory of Medical Rescue Key Technology and Equipment, Ministry of Emergency Management, School of Disaster and Emergency Medicine, Tianjin University, Tianjin, China; 2 Wenzhou Safety (Emergency) Institute of Tianjin University, Wenzhou, Zhejiang, China; 3 State Key Laboratory of Advanced Medical Materials and Devices, School of Medicine, Tianjin University, Tianjin, China; 4 Research Center, Wenzhou People’s Hospital, Wenzhou, Zhejiang, China

**Keywords:** biomarkers, ferroptosis, oxidative stress, smoke inhalation injury, therapeutic targets

## Abstract

Smoke inhalation injury (SII) is a prevalent yet underrecognized cause of acute respiratory failure and longer-term airway-alveolar remodeling after fires, wildfires, and industrial accidents. Beyond inflammation and generic oxidative stress, accumulating evidence supports ferroptosis as a mechanistically distinct, self-amplifying axis that can sustain epithelial/endothelial damage, barrier breakdown, and maladaptive repair. Ferroptosis is driven by iron-dependent phospholipid peroxidation and collapse of membrane redox homeostasis, and in SII is promoted by iron dyshomeostasis including heme/hemoglobin-derived iron and ferritinophagy, PUFA-phospholipid remodeling, and failure of antioxidant checkpoints centered on the System Xc^−^-GSH-GPX4 pathway, with complementary protection via FSP1-CoQ10 and related radical-trapping systems. Despite rapid progress, how ferroptotic signaling integrates with other regulated cell-death programs and inflammatory circuits across the temporal phases of SII remains incompletely defined. In this review, we synthesize molecular mechanisms, crosstalk networks, candidate biomarkers, and preclinical evidence for therapeutically actionable nodes, highlighting ferroptosis inhibitors, iron-targeting approaches, and redox-restorative strategies as candidates for mechanism-guided, disease-modifying intervention in SII.

## 1 Introduction

Smoke inhalation injury (SII) is a major but persistently underestimated cause of morbidity and mortality following fires, wildfires, and industrial accidents worldwide. Exposure to complex mixtures of toxic gases, including carbon monoxide (CO) and hydrogen cyanide (HCN), together with fine particulate matter, induces severe pulmonary injury and accounts for up to 77% of fire-related deaths (Galeiras, 2021; Lobo-Antuña et al., 2026). Despite advances in burn management and critical care, epidemiological studies consistently report that approximately 19.8% of hospitalized burn patients sustain inhalation injury, a proportion that has remained largely unchanged for decades and is strongly associated with increased hospital mortality, particularly in intensive care settings (Witt et al., 2021; Charles et al., 2022). Beyond acute lethality, SII confers a substantial long-term disease burden. Inhaled toxicants impair oxygen delivery via carboxyhemoglobin (COHb) formation and mitochondrial respiratory inhibition, while simultaneously disrupting airway epithelial integrity, endothelial barrier function, and alveolar type II cell survival (Wiegman et al., 2020; He Q. et al., 2024). These processes predispose patients to acute lung injury (ALI) and acute respiratory distress syndrome (ARDS) and drive chronic structural remodeling characterized by fibrosis, emphysema, and irreversible loss of lung function (Niu et al., 2022). Increasing clinical and experimental evidence further establishes SII as a systemic disorder, in which pulmonary injury acts as a central hub initiating lung-to-system inflammatory and metabolic cascades that contribute to chronic cardiovascular dysfunction, neuroinflammation, and cognitive impairment (Williams et al., 2024; Gonzales et al., 2025).

Mechanistic understanding of SII has centered on inflammation and oxidative stress as dominant drivers of tissue injury. Acute smoke exposure triggers rapid recruitment of neutrophils and macrophages, excessive production of reactive oxygen species (ROS), and release of pro-inflammatory cytokines such as Tumor necrosis factor-α (TNF-α), Interleukin-1β (IL-1β), and Interleukin-6 (IL-6), culminating in epithelial and endothelial damage, alveolar flooding, and impaired gas exchange (de Carvalho et al., 2016; Guo et al., 2019). While these processes are indispensable in shaping early injury severity, accumulating evidence indicates that they are insufficient to explain the delayed progression, irreversibility, and multi-organ nature of smoke-induced pathology (Albright et al., 2012). A subset of SII survivors develops progressive pulmonary fibrosis and systemic dysfunction months to years after the initial insult, even in the apparent absence of ongoing inflammation (Yang et al., 2017). Moreover, antioxidant- or anti-inflammatory-based interventions, although effective in attenuating early injury in experimental models, have shown limited success in preventing long-term fibrotic remodeling and extrapulmonary sequelae (Guo et al., 2019; Mercel et al., 2020; Bezerra et al., 2023). These observations underscore a critical gap in current pathophysiological frameworks and point to the involvement of additional, self-sustaining mechanisms that couple acute injury to chronic disease.

SII remains a therapeutically underserved condition. Although it markedly increases burn-associated mortality, with some studies reporting an approximately 20-fold elevation in death risk among burn patients complicated by inhalation injury, current management remains largely supportive in the absence of a definitive pharmacological strategy (Enkhbaatar et al., 2016). Advances in diagnosis and treatment have also lagged behind those achieved in cutaneous thermal injury, highlighting a persistent disconnect between expanding preclinical knowledge and effective clinical translation (Rehberg et al., 2009). A central obstacle to therapeutic development is the pronounced heterogeneity of SII across clinical and biological dimensions. Smoke is not a uniform injurious stimulus; each fire generates a distinct mixture of particulates, respiratory irritants, systemic toxicants, and heat, which together shape the pattern and severity of tissue damage (Walker et al., 2015). At the patient level, differences in exposure intensity, chemical toxicity, smoke temperature, ambient oxygen concentration, and secondary systemic responses may explain why individuals exposed to apparently similar fire environments develop divergent clinical courses (Shubert and Sharma, 2026). Even among patients with comparable exposure histories and bronchoscopic injury grades, the rates of respiratory deterioration, pneumonia, and progression to ARDS vary considerably, suggesting that host biological susceptibility is a major determinant of disease evolution (Amarasekera et al., 2025).

The most pressing unmet need is therefore to identify mechanism-based therapeutic targets that extend beyond supportive care. Despite its substantial contribution to burn morbidity and mortality, SII remains relatively underexplored, with much of the available mechanistic evidence derived from preclinical models (Dyamenahalli et al., 2019). Current pathological frameworks emphasizing oxidative stress, inflammation, and structural airway damage do not fully account for the interpatient variability observed in clinical outcomes. Ferroptosis may provide a biologically plausible explanation for part of this variability, but its role in SII remains insufficiently investigated. For example, patients with extensive hemoglobin degradation, microhemorrhage, or severe glutathione depletion after toxic smoke exposure may exhibit a lower threshold for iron-dependent lipid peroxidation and ferroptotic cell death than those with predominantly airway-restricted injury (Wen et al., 2024). From this perspective, ferroptosis may represent a mechanistic axis linking smoke exposure, redox dysregulation, iron overload, and heterogeneous clinical progression, thereby providing a rationale for evaluating ferroptosis-related pathways as therapeutically actionable targets in SII.

Ferroptosis has recently emerged as a biologically plausible contributor to this unresolved mechanistic landscape. Distinct from apoptosis and necrosis, ferroptosis is an iron-dependent form of regulated cell death driven by uncontrolled lipid peroxidation and failure of cellular redox homeostasis (Yoshida et al., 2019; Xu et al., 2021; Fujii and Imai, 2024; Yang et al., 2025). Dysregulated iron metabolism, depletion of antioxidant defenses, and accumulation of lipid peroxides enable ferroptotic cell death to persist beyond the acute inflammatory phase, providing a plausible explanation for delayed and progressive tissue injury following smoke exposure (Yin et al., 2021; Yang et al., 2025). Pulmonary endothelial and epithelial cells appear particularly vulnerable to ferroptosis, resulting in sustained vascular leakage, pulmonary edema, fibroblast activation, and fibrotic remodeling (Yoshida et al., 2019; Yao et al., 2025b). In addition, ferroptotic cells release damage-associated molecular patterns (DAMPs) that perpetuate immune activation and establish a feed-forward loop linking regulated cell death to chronic inflammation (Wang et al., 2025c; Yao et al., 2025b). Preclinical studies demonstrating protective effects of ferroptosis inhibitors including ferrostatin-1 (Fer-1), liproxstatin-1 (Lip-1), deferoxamine (DFO), and sodium pyruvate, highlight ferroptosis as a therapeutically actionable axis in SII (Yoshida et al., 2019; Zhao et al., 2023; Zhang F. et al., 2024; Zhang H. et al., 2025). Accordingly, this review synthesizes current evidence positioning ferroptosis at the intersection of mechanisms, interventions, and clinical translation in SII, aiming to reframe SII as a disorder of dysregulated cell death and to inform the development of mechanism-based, disease-modifying therapies.

SII is a multifactorial clinicopathological entity in which no single mode of cell death or tissue injury operates in isolation. Established mechanisms, including oxidative stress, airway inflammation, mucosal epithelial apoptosis, neutrophil-mediated pulmonary injury, and surfactant dysfunction, all contribute to disease expression, although their relative weight varies according to smoke composition, exposure intensity, anatomical injury distribution, and host-specific biological susceptibility (Enkhbaatar et al., 2016; Walker et al., 2015). Within this context, ferroptosis should not be framed as a predominant or all-encompassing mechanism, but rather as a context-dependent injury pathway that may amplify, converge with, or mechanistically intersect with established pathological processes. Accordingly, this review aims to define the biological and clinical settings in which ferroptosis is most likely to participate in SII, clarify where it provides explanatory value beyond conventional paradigms, and assess whether ferroptosis-related pathways represent therapeutically tractable targets.

## 2 Molecular mechanisms of ferroptosis

Ferroptosis is a regulated cell-death modality defined by iron-dependent phospholipid peroxidation and collapse of membrane redox homeostasis. Ferroptotic commitment reflects coordinated disruption across key metabolic layers, including expansion of the LIP through altered iron storage/trafficking and ferritinophagy, lipid remodeling that enriches peroxidation-prone Polyunsaturated fatty acid (PUFA)-containing phospholipids (e.g., ACSL4- Lysophosphatidylcholine Acyltransferase 3 (LPCAT3) axis), and failure of antioxidant defense systems that detoxify lipid hydroperoxides. Central protective checkpoints include System Xc^−^-GSH-GPX4 and parallel radical-trapping pathways such as Ferroptosis suppressor protein 1(FSP1)- Coenzyme Q10 (CoQ10). Beyond these core modules, ferroptosis interfaces with apoptosis, necroptosis, pyroptosis, and autophagy via shared stress signaling and convergent execution at the level of oxidative membrane injury. This section focuses on the molecular circuitry of iron handling, lipid peroxidation propagation, antioxidant safeguards, and regulated cell-death crosstalk that collectively govern ferroptosis susceptibility and execution (Figure 1).

**FIGURE 1:**
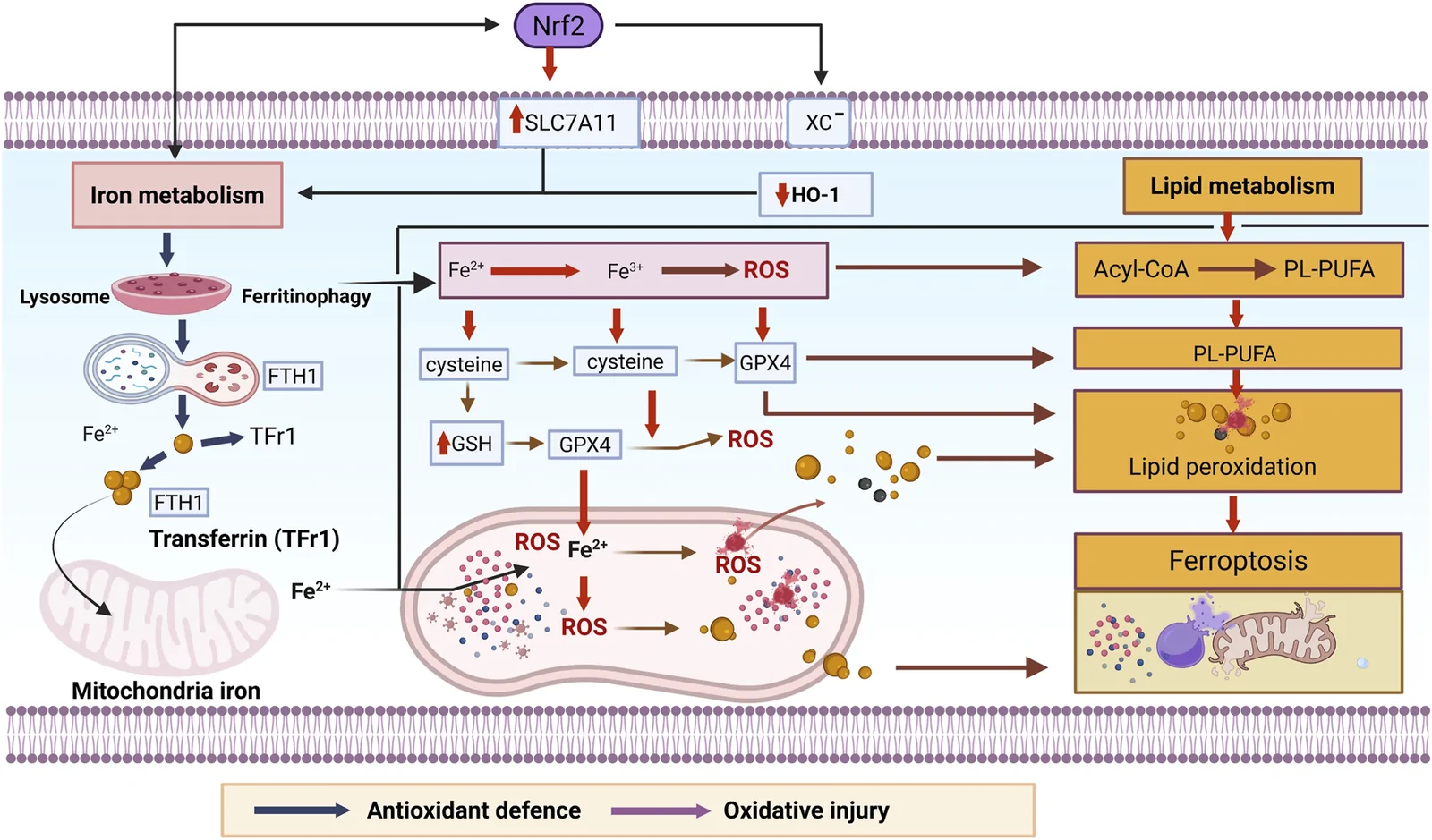
Molecular mechanisms of ferroptosis. Ferroptosis is an iron-dependent form of regulated cell death driven by lipid peroxidation. Intracellular iron homeostasis is regulated via ferritinophagy-mediated degradation of ferritin (FTH1), releasing Fe^2+^, which is transported by transferrin (Tf/TfR1) and accumulates in mitochondria, promoting ROS generation. Excess Fe^2+^ catalyzes Fenton reactions, amplifying ROS production and lipid peroxidation of polyunsaturated phospholipids (PL-PUFA) derived from Acyl-CoA. The cystine/glutamate antiporter (system Xc^−^, SLC7A11), under Nrf2 regulation, imports cystine for GSH synthesis, which in turn supports GPX4 activity to detoxify lipid peroxides and inhibit ferroptosis. Downregulation of GPX4 or GSH depletion, combined with increased ROS from iron overload, drives irreversible lipid peroxidation and ferroptotic cell death. Additional modulators include heme oxygenase-1 (HO-1), which influences iron availability and oxidative stress. The coordinated interplay of iron metabolism, ROS generation, antioxidant defenses, and lipid peroxidation underlies ferroptosis execution in cells.

### 2.1 Iron metabolism imbalance

Ferroptosis is initiated when iron homeostasis is disrupted, allowing redox-active iron to drive lipid peroxide formation and oxidative membrane damage. Because iron is indispensable for mitochondrial respiration, enzymatic catalysis, and cellular metabolism, intracellular iron levels are normally maintained within a tightly regulated range. When this regulatory network fails, ferrous iron (Fe^2+^) accumulates within the LIP, where it catalyzes Fenton chemistry, accelerates phospholipid peroxidation, and progressively compromises the antioxidant systems that restrain ferroptotic cell death (Rochette et al., 2022; Catapano et al., 2025; Ru et al., 2024). Ferroptotic susceptibility is shaped by several converging mechanisms that expand the LIP. Ferritin, composed of ferritin heavy chain 1 (FTH1) and ferritin light chain (FTL), normally sequesters excess iron in a relatively inert form. Under stress conditions, however, ferritin can be selectively delivered to lysosomes through Nuclear Receptor Coactivator 4 (NCOA4)-mediated ferritinophagy, resulting in ferritin degradation and Fe^2+^ release, thereby sensitizing cells to ferroptosis (Zhang X. et al., 2025; Cloonan et al., 2026; Huang et al., 2025; Ning et al., 2026). Inhibition of mTOR signaling further enhances ferritinophagy, linking nutrient-sensing pathways to iron-dependent regulated cell death (Conjard-Duplany et al., 2025; Koshizuka et al., 2025). Mitochondria also act as critical amplification nodes. Oxidative stress-induced mitochondrial injury can promote ferritin degradation, intracellular iron mobilization, and subsequent lipid peroxidation. This mitochondria-ferritinophagy axis has been reported in environmental injury models, including nanoplastic-induced lung damage, suggesting that it may represent a relevant mechanism of tissue injury following inhalational exposure to toxic particles (Yang et al., 2023). Iron influx and efflux systems further determine cellular vulnerability to ferroptosis. Transferrin receptor 1 (TfR1)-mediated iron uptake and ferroportin (FPN)-dependent iron export constitute a major regulatory framework for intracellular iron balance. Disruption of autophagic TfRC degradation, as observed in the context of WD Repeat Domain 45 (WDR45) mutation, can increase iron retention and thereby promote ferroptotic sensitivity. Similarly, upregulation of metal transporters such as Zrt- and Irt-like Protein 14 (ZIP14) enhances intracellular iron accumulation and has been implicated in ferroptosis-associated injury in myocardial ischemia-reperfusion and diabetic nephropathy (Zhao et al., 2026). At the systemic level, the hepcidin-FPN axis governs iron distribution by regulating FPN-mediated iron export; dysregulation of this pathway may therefore promote pathological iron retention and organ injury. Conversely, cells may acquire ferroptosis resistance through FPN-independent iron disposal mechanisms. For example, Prominin2 facilitates iron export via multivesicular bodies and exosome-associated pathways, thereby lowering intracellular iron availability and limiting ferroptotic damage (Brown et al., 2019). Collectively, ferroptosis emerges from the coordinated failure of iron storage, iron trafficking, and iron export systems. Ferritinophagy, mitochondrial dysfunction, altered iron transporter activity, and impaired iron efflux converge to create an iron-overloaded intracellular environment that favors lipid peroxidation and ferroptotic tissue injury. This framework identifies several therapeutically actionable nodes, including ferritin stabilization, inhibition of NCOA4-mediated ferritinophagy, restoration of iron export capacity, and iron chelation. Rather than treating iron dysregulation as a secondary consequence of tissue injury, this perspective positions disordered iron handling as an upstream driver of ferroptosis-associated pathology.

### 2.2 Lipid peroxidation pathways

Lipid peroxidation represents a central biochemical event linking iron dysregulation to ferroptotic injury in SII. Disturbed iron handling, including enhanced TfRC-mediated iron uptake and ferritin degradation, expands the LIP, thereby promoting Fenton chemistry and the generation of lipid radicals that damage biological membranes (Hu et al., 2024; Ru et al., 2024). In the context of smoke exposure, inhaled toxicants, mitochondrial redox imbalance, and iron mobilization may act together to position iron overload as an upstream driver of lipid peroxidation rather than a merely secondary consequence of tissue injury. PUFA-enriched phospholipids within cellular and mitochondrial membranes are the principal substrates for peroxidative damage once intracellular iron availability increases. This process proceeds through two partially overlapping routes. In the enzymatic pathway, Acyl-CoA synthetase long-chain family member 4 (ACSL4) and LPCAT3 incorporate PUFA, particularly arachidonic acid and adrenic acid, into phosphatidylethanolamines, thereby increasing the susceptibility of airway epithelial membranes to ferroptotic injury (Punziano et al., 2024; Zhou et al., 2024b). In parallel, smoke-derived ROS can initiate non-enzymatic oxidation of membrane phospholipids, establishing a feed-forward cycle of lipid radical formation, membrane destabilization, and alveolar barrier disruption (Upadhyay et al., 2024). Among the counter-regulatory systems, the GSH-GPX4 axis constitutes a major protective checkpoint. In SII, GSH depletion, suppression of system Xc^−^ activity through Solute Carrier Family 7 Member 1 (SLC7A11) impairment, and loss of GPX4 function may collectively reduce phospholipid hydroperoxide detoxification, allowing lipid peroxides to accumulate beyond the threshold for membrane failure. Nuclear factor erythroid 2-related factor 2 (Nrf2) occupies a pivotal regulatory position in this network by coupling oxidative stress sensing to the transcriptional induction of antioxidant enzymes, cystine transport machinery, and iron-sequestering proteins, making the Nrf2-GPX4 axis a rational therapeutic target (Li et al., 2022; Gao et al., 2025). The terminal phase of ferroptotic injury is characterized by catastrophic membrane damage caused by unchecked lipid peroxide accumulation after antioxidant defenses are exhausted. This mechanism provides a plausible explanation for the extensive epithelial injury, vascular leakage, persistent inflammation, and subsequent fibrotic remodeling observed in severe SII. It also defines several therapeutic interception points, including direct ferroptosis inhibitors, iron chelators such as DFO, and strategies aimed at restoring Nrf2-GPX4-dependent antioxidant capacity (Yan et al., 2021). Evidence from SII-related experimental models and smoke-exposure studies suggests that targeting these nodes can reduce epithelial inflammation, limit lipid ROS accumulation, and preserve alveolar architecture. Notably, some traditional Chinese medicine-derived compounds may exert dual regulatory effects on iron handling and lipid peroxidation, supporting their potential relevance as multi-target interventions in ferroptosis-associated smoke injury (Xu M. et al., 2024). This framework reframes SII not only as an inflammatory airway disorder, but also as a lipid peroxidation-driven injury state, thereby shifting therapeutic emphasis from nonspecific respiratory support toward mechanism-based molecular intervention.

### 2.3 Antioxidant defense systems and ferroptosis regulation

Ferroptosis is constrained by three major antioxidant defense modules: system Xc^−^, the GSH-GPX4 axis, and the CoQ10-FSP1 pathway, which together provide layered protection against phospholipid peroxide accumulation (Chen F. et al., 2024; Alves et al., 2025). Conceptually, ferroptosis reflects the collapse of these protective systems, culminating in uncontrolled peroxidation of PUFA-containing phospholipids. Cell fate is therefore determined by the dynamic balance between lipid peroxide-generating execution mechanisms and the residual capacity of antioxidant defenses. Each module operates at a distinct regulatory level and may display differential vulnerability to toxic insults associated with SII. System Xc^−^ functions as an upstream regulatory checkpoint by controlling intracellular cysteine availability. This heterodimeric antiporter, composed of the catalytic subunit SLC7A11/xCT and the chaperone subunit SLC3A2, mediates the exchange of extracellular cystine for intracellular glutamate. Imported cystine is subsequently reduced to cysteine, the rate-limiting precursor for GSH biosynthesis (Parker et al., 2021). Because GPX4 requires adequate GSH to detoxify phospholipid hydroperoxides, disruption of system Xc^−^ reduces GSH synthesis, impairs GPX4 activity, and weakens resistance to ferroptosis. Conversely, SLC7A11 upregulation can protect cells from iron-driven lipid peroxidation. This node is also shaped by immune and stress-response signals. For example, interferon-γ released by CD8^+^ T cells can suppress SLC3A2 and SLC7A11, thereby promoting ferroptosis in tumor cells, whereas activation of system Xc^−^ protects macrophages from ferroptotic death (Xia et al., 2025). The stress-responsive transcription factor Activating Transcription Factor 3 (ATF3) further links cellular stress to antioxidant failure by transcriptionally repressing SLC7A11. These observations position system Xc^−^ as a mechanistically important and potentially druggable upstream determinant of ferroptosis sensitivity in SII.

GPX4 represents the principal enzymatic barrier against ferroptosis by reducing phospholipid hydroperoxides to non-toxic lipid alcohols in a GSH-dependent manner. Beyond its canonical anti-ferroptotic function, GPX4 contributes to phospholipid homeostasis, embryonic development, and cell survival. However, pharmacological or genetic inhibition of GPX4 rapidly induces lipid peroxide accumulation and ferroptotic cell death (Vučković et al., 2020). GPX4 activity is additionally controlled by post-translational mechanisms that influence protein stability. The deubiquitinase USP8, for instance, protects GPX4 from proteasomal degradation and thereby contributes to ferroptosis resistance and tumor immune regulation (Li et al., 2024). Nevertheless, GPX4 depletion does not uniformly predict ferroptotic outcome, as nuclear factor kappa B (NF-κB) activation can engage compensatory protective programs that reshape the cell-death response (Jin et al., 2024). Post-transcriptional regulation also modulates this axis; miR-15a-3p suppresses GPX4 expression, increasing ROS generation and lipid peroxidation (Liu et al., 2022). Together, these findings indicate that GPX4 functions not as an isolated endpoint, but as a dynamically regulated hub embedded within broader stress-response and immune-regulatory networks.

The FSP1-CoQ10 pathway constitutes a major GPX4-independent ferroptosis defense system. At the plasma membrane, FSP1 catalyzes the NAD(P)H-dependent reduction of ubiquinone (CoQ10) to ubiquinol (CoQ10H2), a lipophilic radical-trapping antioxidant that limits lipid peroxidation (Bersuker et al., 2019). Acting in parallel with the GSH-GPX4 system, FSP1 provides an additional protective layer against ferroptotic membrane injury and has emerged as a potential therapeutic target in ferroptosis-sensitive contexts (Doll et al., 2019). Its activity is regulated by Nrf2-mediated transcriptional induction, vitamin B2/FAD-dependent stabilization, and ubiquitination-related control by RNF8 and TRIM21, which collectively influence FSP1 stability, membrane localization, and anti-ferroptotic capacity (Koppula et al., 2022; Deol et al., 2025; Gong et al., 2023). Thus, FSP1-dependent defense may be particularly relevant when GPX4 activity is compromised in SII-associated ferroptotic injury.

Collectively, these antioxidant defense systems form a hierarchical and redundant network that determines ferroptosis susceptibility. From a mechanistic standpoint, ferroptosis emerges not as the consequence of a single molecular lesion but as a systems-level collapse of lipid peroxide detoxification capacity. This integrated perspective is essential for interpreting ferroptosis signaling diagrams and for rationally designing therapeutic strategies that restore redox balance in ferroptosis-driven pathologies.

### 2.4 Cell-type-specific mechanisms of ferroptosis in the lung

Previous studies of ferroptosis in lung injury have often treated the lung as a relatively uniform target of oxidative and iron-dependent stress. However, accumulating evidence indicates that ferroptotic susceptibility and defense mechanisms differ substantially among pulmonary cell populations. In particular, alveolar epithelial type II (AT2) cells and alveolar macrophages (AMs) rely on distinct ferroptosis-regulatory modules, reflecting their divergent biological functions, redox states, metabolic programs, and iron-handling capacities. AT2 cells primarily depend on epithelial antioxidant systems to preserve alveolar integrity, whereas AMs must accommodate a higher iron burden because of their roles as professional phagocytes, immune sentinels, and iron-recycling cells. Recognizing this cellular heterogeneity is essential for refining the mechanistic interpretation of ferroptosis-related lung pathology and for developing interventions that target ferroptosis in a cell-context-dependent manner.

#### 2.4.1 AT2 cells: GPX4-centric defense, epigenetic repression, and NCOA4-mediated ferritinophagy

AT2 cells are indispensable for alveolar homeostasis, surfactant production, epithelial repair, and cytokine-mediated regulation of the distal lung microenvironment. Their functional status is closely linked to the initiation and progression of acute and chronic lung injury. Because AT2 cells are metabolically active and constantly exposed to oxidative and inflammatory stress within the alveolar niche, they are particularly dependent on robust antioxidant systems to prevent phospholipid peroxidation and ferroptotic cell death. Within this context, the GPX4-dependent antioxidant system represents a central protective mechanism, as GPX4 detoxifies phospholipid hydroperoxides and prevents the propagation of lipid peroxidation (Sun et al., 2025). This GPX4-centered defense is embedded within a broader antioxidant transcriptional network, in which NRF2/Keap1 signaling plays a major regulatory role. NRF2 functions as a master transcriptional regulator of antioxidant and cytoprotective genes, including those involved in GSH metabolism, iron handling, and lipid peroxide detoxification. Upon oxidative stress, NRF2 translocates into the nucleus and activates antioxidant gene programs that restore redox balance. Conversely, NRF2 deficiency aggravates lung inflammation and tissue injury in pulmonary fibrosis models, whereas pharmacological activation of NRF2 mitigates oxidative damage by reinforcing endogenous antioxidant defenses (Liu, et al., 2025). Therefore, insufficient NRF2 activation may simultaneously weaken multiple ferroptosis-suppressive pathways and lower the threshold for epithelial ferroptotic injury.

Emerging evidence further suggests that AT2-cell susceptibility to ferroptosis is not governed solely by acute oxidative stress, but is also shaped by epigenetic repression of ferroptosis-suppressive genes. During pulmonary fibrosis progression, ferroptosis of alveolar epithelial cells is increased, whereas the ferroptosis suppressor genes GPX4 and FSP1 are transcriptionally silenced. Mechanistically, the methylation regulator Ubiquitin-like with PHD and RING Finger Domains 1 (UHRF1) promotes CpG methylation within the GPX4 and FSP1 promoters, thereby suppressing two major ferroptosis-defense modules. Pharmacological or genetic inhibition of UHRF1 attenuates ferroptosis and limits fibrotic progression in experimental models (Liu et al., 2022). This mechanism is particularly relevant because FSP1 acts as a GSH-independent ferroptosis suppressor that operates in parallel with GPX4; its N-terminal myristoylation is required for plasma membrane localization, where it inhibits lipid peroxidation-driven ferroptotic death (Doll et al., 2019). Thus, UHRF1-mediated co-repression of GPX4 and FSP1 may simultaneously compromise two complementary anti-ferroptotic barriers, rendering AT2 cells especially vulnerable to oxidative and electrophilic stress.

Beyond the repression of antioxidant defenses, dysregulated iron mobilization constitutes another major mechanism driving AT2-cell ferroptosis. In ALI, ferroptotic AT2 cells display characteristic morphological and biochemical alterations, including mitochondrial shrinkage, mitochondrial membrane disruption, iron overload, GSH depletion, and GPX4 downregulation (Li W. et al., 2025). NCOA4-mediated ferritinophagy provides a direct mechanistic link between iron mobilization and ferroptosis. As a selective autophagic process, ferritinophagy delivers ferritin to autophagosomes for degradation, thereby releasing ferritin-bound iron into the cytosol, expanding the LIP, and accelerating Fenton chemistry and lipid peroxide formation (Yao et al., 2025b). Recent mechanistic studies have demonstrated that NCOA4-dependent ferritinophagy is functionally active in alveolar epithelial cells. The ANG II/AGTR1 axis can induce ferritinophagy, resulting in iron accumulation and ferroptotic injury, whereas mechanical stress promotes NCOA4-associated ferritin phase separation and ferritinophagic degradation in alveolar epithelial cells (Zhu et al., 2025). These findings indicate that AT2-cell ferroptosis may arise from the convergence of impaired antioxidant defense, epigenetic repression of GPX4/FSP1, and ferritinophagy-driven iron mobilization.

#### 2.4.2 AMs: FSP1/CoQ10, NCOA4-STING-mediated ferritinophagy, and polarization-dependent ferroptosis regulation

AMs face a ferroptotic burden distinct from that of AT2 cells because of their dual role as professional phagocytes and iron-recycling immune cells. By internalizing apoptotic cells, erythrocyte-derived material, particulates, and extracellular debris, AMs are frequently exposed to heme and iron-rich substrates. Heme oxygenase-1 (HO-1), which is induced by heme, oxidative stress, inflammation, and infection, degrades heme into biliverdin, carbon monoxide, and ferrous iron. Biliverdin is rapidly converted into bilirubin and exerts antioxidant and anti-inflammatory effects, whereas Fe^2+^ expansion of the LIP may accelerate lipid peroxidation. This duality positions HO-1 as both a cytoprotective stress-response enzyme and a potential amplifier of ferroptosis under conditions of sustained iron loading (Zhou et al., 2025). Recent evidence further suggests that limiting macrophage ferroptosis can attenuate lung inflammatory injury, supporting the view that ferroptotic AMs are not merely passive targets of tissue stress but active contributors to inflammatory damage. Unlike AT2 cells, in which GPX4-centered antioxidant defense is often emphasized, AM ferroptosis may be more dependent on the FSP1/CoQ10 axis. FSP1 restrains lipid peroxidation independently of the canonical GSH-GPX4 pathway by functioning as an NADPH-dependent oxidoreductase that regenerates reduced CoQ10, a lipophilic radical-trapping antioxidant at cellular membranes (Zhu et al., 2025; Li W. et al., 2025). The anti-ferroptotic activity of FSP1 requires N-terminal myristoylation, which directs FSP1 to membrane-associated compartments, including the plasma membrane, Golgi region, and perinuclear membranes; mutation of this myristoylation site abolishes its ferroptosis-suppressive function (Zhang W. et al., 2024). Although GPX4 remains highly relevant in chronic lung diseases such as Chronic obstructive pulmonary disease (COPD), emerging evidence indicates that FSP1 can functionally cooperate with GPX4 in pulmonary disease contexts, suggesting that macrophage ferroptosis is governed by a layered antioxidant network rather than by a single linear pathway (Yang et al., 2025).

In parallel, NCOA4-mediated ferritinophagy provides another critical layer linking macrophage iron turnover to inflammatory injury. As an NCOA4-dependent selective autophagic process, ferritinophagy degrades ferritin and mobilizes stored iron, thereby increasing the LIP available for ferroptotic lipid peroxidation. Beyond iron release, this pathway intersects with immune-regulatory signaling networks, including Mitogen-Activated Protein Kinase (MAPK), cGAS-Stimulator of interferon genes (STING), NF-κB, AMPK/mTOR, and NRF2, placing ferritinophagy at the interface between iron metabolism, oxidative stress, and innate immune activation (Sahu and Jain, 2025). In macrophages, STING signaling can directly engage this axis: activation of cGAS-STING and interferon-associated pathways promotes macrophage ferroptosis, while STING has been shown to bind cytoplasmic NCOA4 and facilitate ferritin degradation, free Fe^2+^ release, and lipid peroxidation (Wu et al., 2022). The STING-NCOA4-ferritinophagy axis may therefore constitute a mechanistic bridge between innate immune sensing, iron mobilization, and macrophage ferroptotic remodeling.

AM ferroptosis is also dynamically regulated by macrophage polarization state. M1-like macrophage activation, particularly under TNF-α and IFN-γ stimulation, induces nitric oxide (NO) production and can enhance RIPK3/MLKL-mediated necroptosis and tissue injury (Zheng et al., 2023). However, this inflammatory state may also confer a relative ferroptosis-resistant phenotype, because iNOS-derived nitric oxide can prevent Arachidonate 15-Lipoxygenase (ALOX15) from accessing lipid radical substrates, thereby limiting lipid peroxidation. Experimental evidence further indicates that macrophage-derived NO can suppress phospholipid peroxidation in AT2 cells, and this protection persists even when epithelial GPX4 is genetically reduced, suggesting a GPX4-independent anti-ferroptotic mechanism (Dar et al., 2021). Thus, M1-associated NO production may function as both an intrinsic macrophage ferroptosis-modulating mechanism and a paracrine protective signal for neighboring epithelial cells. By contrast, M2-like macrophages rely more heavily on oxidative phosphorylation and fatty acid β-oxidation and exhibit increased FPN expression, metabolic features that may reshape intracellular iron handling and cell-death susceptibility. Given that ferroptosis is driven by iron-dependent lipid peroxidation and is shaped by oxidative stress and local inflammatory cues (Shen et al., 2025), transitions in macrophage activation state are likely to create distinct windows of ferroptotic vulnerability. Early M1 polarization may transiently protect AMs from ferroptosis through iNOS-dependent mechanisms, whereas sustained IL-4/IL-13-driven M2 polarization may weaken this protection and increase susceptibility to ferroptotic injury. Therefore, ferroptosis in AMs is not determined solely by iron load or antioxidant capacity, but by the integration of immune polarization, iron export, mitochondrial metabolism, nitric oxide signaling, and ferritinophagy-associated innate immune activation.

Taken together, AT2 cells and AMs display divergent but interconnected ferroptosis-regulatory programs. Epithelial ferroptosis is mainly linked to the collapse of GPX4/FSP1-associated antioxidant defenses, NRF2 insufficiency, epigenetic repression, and NCOA4-mediated iron mobilization. In contrast, macrophage ferroptosis is more dynamically coupled to phagocytic iron burden, HO-1 activity, FSP1/CoQ10-dependent membrane protection, STING-NCOA4-mediated ferritinophagy, polarization-dependent metabolism, and paracrine NO signaling. This AT2-AM contrast reinforces the concept that ferroptosis-targeted interventions should be tailored to cell-type-specific vulnerability rather than applied as a uniform therapeutic strategy (Figure 2).

**FIGURE 2:**
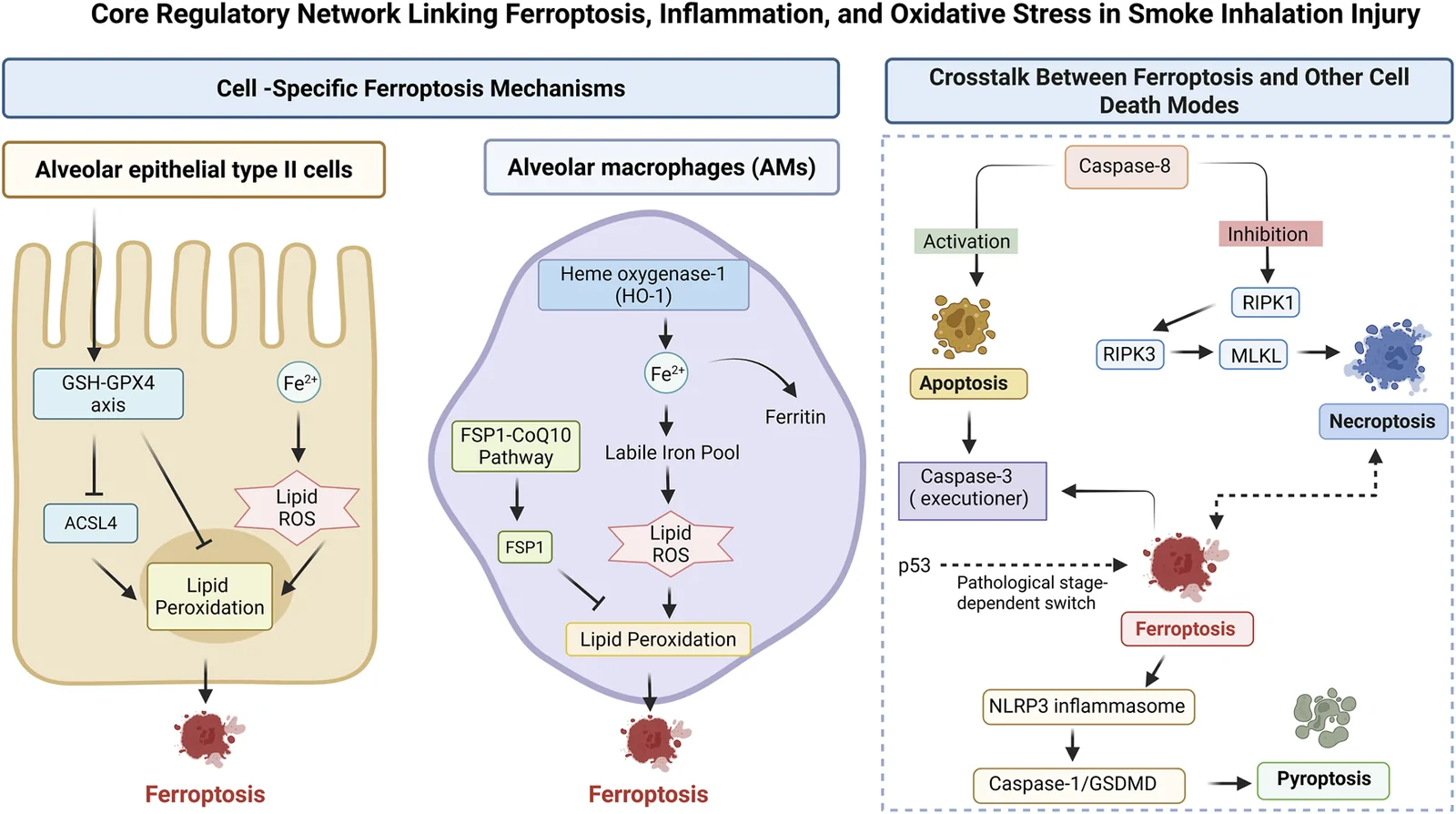
Core regulatory network linking ferroptosis, inflammation, and oxidative stress in smoke inhalation injury. This schematic depicts cell-specific ferroptotic regulatory programs in alveolar type II epithelial cells (AT2) and alveolar macrophages (AMs), together with the intersection between ferroptosis and other regulated cell-death pathways. In AT2 cells, ferroptotic susceptibility is primarily shaped by disruption of the GSH-GPX4 antioxidant axis. GPX4 inactivation, ACSL4-driven phospholipid remodeling, iron accumulation, and ROS production converge to promote lipid peroxidation and ferroptotic membrane injury. By contrast, AMs appear more dependent on iron-handling and redox-buffering pathways, including ferritin turnover, expansion of the labile iron pool, heme oxygenase-1 activity, and the FSP1-mediated antioxidant system. The figure also illustrates the molecular crosstalk between ferroptosis and apoptosis, necroptosis, and pyroptosis through key regulators such as p53, caspase-8, caspase-3, and the NLRP3 inflammasome. Oxidative stress and inflammatory signaling increase ferroptotic sensitivity, whereas ferroptotic injury reciprocally amplifies inflammatory responses, thereby forming a self-reinforcing injury circuit that contributes to the progression of smoke inhalation injury.

### 2.5 Crosstalk between ferroptosis and other forms of cell death

#### 2.5.1 An integrated cell death network centered on ferroptotic lipid peroxidation

Ferroptosis is defined by iron-dependent lipid peroxidation of polyunsaturated phospholipids, culminating in catastrophic membrane damage. Increasing evidence indicates that this core iron-lipid peroxidation module is not isolated but instead interfaces extensively with apoptosis, necroptosis, pyroptosis, and autophagy, forming a network of regulated cell death pathways that converge on oxidative membrane injury. In many pathological contexts, these modes of cell death cannot be strictly segregated and may either counterbalance or reinforce one another, depending on the molecular checkpoints engaged (Chen et al., 2021). To clarify the shared and distinct biochemical triggers, execution mechanisms, and morphologic features across these regulated cell death programs, we provide a concise comparison in Table 1.

**TABLE 1:** Comparison of ferroptosis and other forms of cell death.

Cell death modality	Defining hallmark	Key regulators or readouts	Canonical rescue or inhibition	Role of it in SII or ALI
Ferroptosis	Fe^2+^-dependent phospholipid peroxidation with failure of antioxidant defense	Disturbance of iron metabolism; system Xc^−^-glutathione-GPX4 axis; FSP1-CoQ10 pathway	Fer-1; Lip-1; DFO; sodium pyruvate	The ferroptosis-inflammation positive feedback loop is the core injury mechanism underlying SII and ALI
Apoptosis	Caspase-mediated, genetically regulated programmed cell death	Caspase-3/7; BAX/BAK; p53-CHOP/PUMA	Pan-caspase inhibition (z-VAD-fmk)	It is involved in the loss of epithelial or endothelial cells in SII and ALI, and synergistically exacerbates lung injury
Necroptosis	A form of programmed necrosis characterized by plasma membrane destabilization and mitochondrial dysfunction	RIPK1-RIPK3-MLKL; HSP90	RIPK1 inhibitors; nigratine	Strongly pro-inflammatory; mitochondrial oxidative stress amplify necroptosis in ALI
Pyroptosis	Inflammasome-gasdermin pore formation leading to inflammatory lysis	Inflammasome and caspase 1 activation leads to GSDMD	Inflammasome/caspase-1/GSDMD axis inhibition	Highly inflammatory; relevant to ALI phenotypes through innate immune-autophagy coupling
Autophagy/Ferritinophagy	Selective autophagy promotes ferroptosis by degrading ferritin to release Fe^2+^	mTOR inhibition	OR modulation	Mechanistic bridge in multi-death networks (innate immunity/autophagy/ferroptosis) rather than a standalone endpoint

Apoptosis-ferroptosis crosstalk occurs upstream and downstream of lipid peroxidation. The tumor suppressor tumor protein (p53) occupies a central nodal position in signaling diagrams, as it can transcriptionally regulate both apoptotic mediators and ferroptosis-related genes that influence redox balance and lipid peroxidation susceptibility (Xu et al., 2023). Apoptosis-inducing factor (AIF), classically associated with caspase-independent apoptosis, has been shown to translocate to the nucleus during ferroptosis, linking mitochondrial dysfunction to oxidative DNA damage downstream of lipid peroxidation (Nguyen et al., 2025). Members of the BCL-2 family, long recognized as arbiters of mitochondrial outer membrane permeabilization in apoptosis, also modulate ferroptosis sensitivity. Pharmacological or genetic inhibition of BCL-2 promotes ferroptosis, while excessive ferroptotic lipid peroxidation can suppress apoptotic execution, suggesting a largely unidirectional regulatory relationship at the level of mitochondrial redox homeostasis (Qiu et al., 2025).

Organelle stress signaling integrates ferroptosis with apoptosis through lipid peroxidation-driven endoplasmic reticulum (ER) stress. The ER, mitochondria, and lysosomes function as decision-making hubs in ferroptosis diagrams, coordinating redox stress and cell fate. Ferroptosis inducers such as erastin and artesunate (ART) trigger ER stress responses that amplify lipid peroxidation signaling. Specifically, these agents activate the CHOP pathway, leading to upregulation of the pro-apoptotic protein PUMA independently of p53 (Hong et al., 2017). Loss of therapeutic synergy between erastin, ART, and TRAIL in PUMA-deficient or CHOP-deficient cells but not in p53-deficient cells demonstrates that a CHOP–PUMA axis bridges ferroptotic oxidative stress with death receptor-mediated apoptosis (Lee et al., 2018). Bid acts as a critical molecular switch at this interface: co-treatment with ferroptotic agents and TRAIL enhances caspase-8 activation and Bid truncation, thereby linking lipid peroxidation-induced ER stress to mitochondrial apoptotic amplification (Kim et al., 2022).

Ferroptosis-necroptosis interactions converge on mitochondrial dysfunction and membrane rupture. Necroptosis, a regulated form of necrosis mediated by RIPK1, RIPK3, and MLKL, shares downstream features with ferroptosis, particularly plasma membrane destabilization. Iron overload, as a classical trigger of ferroptosis, can induce mitochondrial permeability transition pore (MPTP) opening, which enhances RIPK1 phosphorylation and promotes necroptotic signaling (Zhou et al., 2021). HSP90 serves as a shared upstream regulator by facilitating RIPK1 activation while simultaneously suppressing GPX4 activity, thereby intensifying lipid peroxidation and ferroptotic execution. These findings place necroptosis and ferroptosis in parallel branches downstream of mitochondrial oxidative stress, capable of cooperating or compensating depending on the inhibitory constraints imposed. The dual inhibitor nigratine exemplifies this overlap, simultaneously attenuating ferroptotic lipid peroxidation and necroptotic kinase activation (Wu et al., 2019).

Pyroptosis and autophagy intersect ferroptosis at the level of iron availability and lipid peroxidation amplification. In inflammatory settings, the cGAS-STING pathway emerges as a unifying regulator that promotes both ferroptosis and pyroptosis. Activation of cGAS-STING induces ferritinophagy, an autophagic process that degrades ferritin and liberates redox-active iron. The resulting expansion of the LIP accelerates phospholipid peroxidation, a hallmark step in ferroptosis diagrams (Hino et al., 2025). Thus, ferritinophagy functions as a mechanistic bridge linking innate immune signaling, autophagy, and ferroptotic membrane damage. The extensive overlap among ferroptosis, necroptosis, and pyroptosis has attracted considerable attention in cancer biology, particularly in the context of immunogenic cell death and antitumor immunity (Liu Y. C. et al., 2024).

Recent integrative models propose that ferroptosis, necroptosis, and pyroptosis collectively shape disease progression in gastrointestinal malignancies and inflammatory disorders (Zhu and Li, 2023). Moreover, host-microbiome interactions can modulate ferroptosis by regulating iron metabolism, mitochondrial function, and lipid peroxidation capacity, further emphasizing the centrality of the ferroptotic redox axis (Ding et al., 2025). Collectively, these observations highlight that lipid peroxidation is a shared execution platform onto which multiple regulated cell death pathways converge, suggesting that therapeutic strategies targeting ferroptosis may exert broader effects by reshaping the entire cell death network.

#### 2.5.2 p53 as a context-dependent bidirectional switch between ferroptosis and apoptosis

In the smoke-injured lung, p53 functions as a context-dependent molecular switch that coordinates ferroptosis and apoptosis according to injury severity, cell identity, and post-translational modification status. Rather than acting through a single linear pathway, p53 can promote ferroptosis through several convergent mechanisms. At the level of cystine metabolism, p53 transcriptionally represses SLC7A11, which encodes the xCT subunit of System Xc^−^, thereby limiting cystine uptake, GSH synthesis, and GPX4-dependent lipid peroxide detoxification. At the level of lipid oxidation, p53 induces SAT1 expression, which in turn upregulates ALOX15 and promotes phospholipid peroxidation through a pathway that can operate partly independently of GPX4 inhibition (Xu et al., 2023). p53 also intersects with lipid substrate availability and mitochondrial iron handling. ACSL4 and LPCAT3 enrich cellular membranes with polyunsaturated fatty acyl-containing phospholipids, which are highly susceptible to enzymatic and non-enzymatic peroxidation. In parallel, iron serves as a catalytic cofactor for ALOX-mediated lipid oxidation and fuels non-enzymatic Fenton chemistry. By increasing the expression of mitochondrial iron-related regulators such as SLC25A28 and FDXR, p53 may further expand the pool of redox-active labile iron and thereby amplify ferroptotic signaling (Wang et al., 2023; Xu et al., 2023; Zhan et al., 2024). Importantly, p53 does not invariably drive ferroptosis. Under conditions of limited or repairable injury, wild-type p53 can exert anti-ferroptotic effects by restricting DPP4 membrane-associated pro-ferroptotic activity and inducing p21, thereby preserving GSH homeostasis and sustaining GPX4 function (Xu et al., 2023; Zhan et al., 2024). This bidirectional behavior suggests that p53 acts less as a fixed executor of cell death than as a stress-adaptive decision node. In severely damaged or irreversibly injured cells, p53-dependent suppression of SLC7A11, activation of the Spermidine/Spermine N^1^-Acetyltransferase 1 (SAT1)/ALOX15 axis, and enhancement of mitochondrial iron stress may favor ferroptosis or cooperate with apoptotic programs to eliminate dysfunctional cells. Conversely, in epithelial progenitor-like or repair-competent cells, particularly AT2 cells required for alveolar regeneration, p53-mediated DPP4 sequestration and p21 induction may prevent excessive ferroptotic loss and preserve tissue-repair capacity. In SII, this regulatory duality is likely to vary across disease stages: the acute oxidative phase may bias p53 output toward SLC7A11 repression and SAT1/ALOX15-driven lipid peroxidation, whereas the subsequent repair phase may favor DPP4 restraint and p21-dependent cytoprotection. Thus, p53 should be interpreted as a context-sensitive rheostat that calibrates ferroptosis-apoptosis balance rather than as a unidirectional pro-death factor.

#### 2.5.3 The NLRP3 inflammasome: bidirectional ferroptosis-pyroptosis regulation

The NOD-like Receptor Family Pyrin Domain Containing 3 (NLRP3) inflammasome represents a central interface through which ferroptosis and pyroptosis are reciprocally regulated in the smoke-injured lung. Evidence from lethal polymicrobial sepsis indicates that excessive lipid peroxidation can promote Gasdermin D (GSDMD)-dependent pyroptosis: conditional deletion of GPX4 increases lipid peroxidation, enhances caspase-11 activation, and augments GSDMD cleavage, suggesting that GPX4 restrains pyroptotic signaling by limiting lipid peroxide accumulation (Zheng et al., 2023). In the alveolar microenvironment of SII, ferroptotic AT2 cells may release DAMPs, including High-mobility group box 1 (HMGB1) and oxidized phospholipids. These signals can activate NLRP3 in adjacent AM’s, leading to caspase-1 activation, GSDMD cleavage, pro-IL-1β/pro-IL-18 maturation, and subsequent pyroptotic cytokine amplification (Shen et al., 2025). Consistent with this ferroptosis-pyroptosis coupling, both forms of regulated cell death contribute to cecal ligation and puncture (CLP)-induced ALI, whereas aldehyde dehydrogenase 2 appears to confer protection by attenuating both processes (Cao et al., 2022). This interaction is not simply linear; it also contains an intrinsic negative-feedback component. The lipid peroxidation-derived aldehyde 4-hydroxynonenal (4-HNE), although generally considered a marker and mediator of oxidative lipid damage, can paradoxically suppress NLRP3 activation by directly binding to the NEK7-interaction domain of NLRP3. This interaction prevents NEK7 recruitment and disrupts inflammasome assembly, with Nrf2- and NF-κB-related signaling further shaping the inflammatory output (Hsu et al., 2022). GPX4 deficiency or pharmacological inhibition increases endogenous 4-HNE accumulation in both *in vivo* and *in vitro* systems, suggesting that lipid peroxidation may generate a self-limiting brake on inflammasome activation under certain conditions. In SII, this mechanism may operate as a threshold-dependent regulatory circuit: moderate ferroptotic lipid peroxidation may produce sufficient 4-HNE to restrain NLRP3 activation, whereas severe oxidative burden caused by intense smoke exposure may exceed this compensatory range, allowing unchecked inflammasome activation, pyroptotic cell death, and cytokine storm amplification. This dose- and context-dependent transition from feedback inhibition to inflammatory escalation may constitute a critical mechanistic threshold in SII pathophysiology (Zhang J. et al., 2024).

#### 2.5.4 Ferroptosis-necroptosis crosstalk: reciprocal antagonism at the ACSL4/MLKL node

Ferroptosis and necroptosis intersect at the level of membrane lipid remodeling, oxidative stress, and regulated membrane rupture, with ACSL4 and MLKL representing two functionally opposed but mechanistically connected nodes. ACSL4 and LPCAT3 promote the incorporation of polyunsaturated fatty acyl chains into membrane phospholipids, generating PUFA-containing phospholipids that are highly susceptible to peroxidation. These substrates can be oxidized through both non-enzymatic and enzymatic mechanisms: iron catalyzes Fenton chemistry to generate lipid radicals, while also serving as an essential cofactor for ALOX enzymes that drive enzymatic phospholipid oxidation. In this way, ACSL4-dependent PUFA-PL enrichment directly couples membrane composition to ferroptotic vulnerability and subsequent membrane destabilization (Wang et al., 2023). Necroptosis injures the alveolar epithelium through a distinct but convergent membrane-disruptive mechanism. Following PAMP- or DAMP-mediated stimulation, the RIPK1-RIPK3-MLKL axis is activated, and RIPK3 phosphorylates MLKL. Phosphorylated MLKL then oligomerizes and translocates to the plasma membrane, where it forms membrane-disrupting pores that compromise epithelial integrity and aggravate alveolar–capillary barrier dysfunction (Kang et al., 2025). The interaction between ferroptosis and necroptosis, however, is not merely additive. Experimental evidence suggests a reciprocal antagonistic relationship: pharmacological suppression of ferroptosis can progressively sensitize cells to necroptosis in a concentration- and time-dependent manner, whereas MLKL deletion increases ferroptotic susceptibility. In MLKL-deficient animals, elevated ACSL4 expression has been observed within 24 h after reperfusion, supporting the concept that blocking the necroptotic machinery may redirect cellular vulnerability toward ferroptotic lipid peroxidation (Müller et al., 2017). Mitochondrial ROS provides an additional point of convergence between these two death programs. Beyond promoting lipid peroxidation, mitochondrial ROS can initiate RIPK1 autophosphorylation and facilitate RIPK3-dependent necrosome assembly, thereby linking oxidative stress to necroptotic execution (Zheng et al., 2023). In the context of SII, this reciprocal ACSL4-MLKL toggle has important therapeutic implications. Selective inhibition of ferroptosis alone may not reduce overall epithelial loss if the injured tissue is simultaneously shifted toward necroptosis. Conversely, isolated blockade of necroptosis may enhance ferroptotic susceptibility through ACSL4-associated lipid remodeling. Therefore, effective cytoprotection in SII may require coordinated intervention across ferroptotic, necroptotic, and inflammatory death pathways, rather than single-node inhibition. Multi-target strategies directed against shared oxidative and inflammatory hubs such as ACSL4, RIPK3, GSDMD, and UHRF1, may offer a more rational framework for limiting alveolar epithelial damage and preserving barrier integrity in the smoke-injured lung.

## 3 Pathological features of SII

SII results from combined thermal stress and toxicants, with CO, HCN, and fine or ultrafine particulate matter acting as major early determinants in a dose and time dependent manner. CO reduces oxygen delivery and inhibits mitochondrial respiration, HCN blocks cellular oxygen utilization, and particulates deposit in distal airspaces to provoke epithelial injury and inflammation with potential systemic spillover. These insults rapidly cause airway and alveolar damage, including epithelial necrosis and sloughing, vascular leakage and edema, mucus hypersecretion with pseudomembrane formation, impaired mucociliary clearance, and barrier failure that increase the risks of obstruction and infection. Persistent oxidative stress amplifies injury through ROS and Reactive nitrogen species (RNS) driven DNA damage, Poly (ADP-ribose) polymerase (PARP) related energy depletion, and mitochondrial dysfunction. In parallel, strong immune activation, marked by neutrophil influx, cytokine and complement surges, and macrophage and dendritic cell reprogramming with impaired adaptive immunity, reinforces a feed forward oxidative and inflammatory cycle that worsens acute injury and contributes to long term pulmonary dysfunction (Figure 3).

**FIGURE 3:**
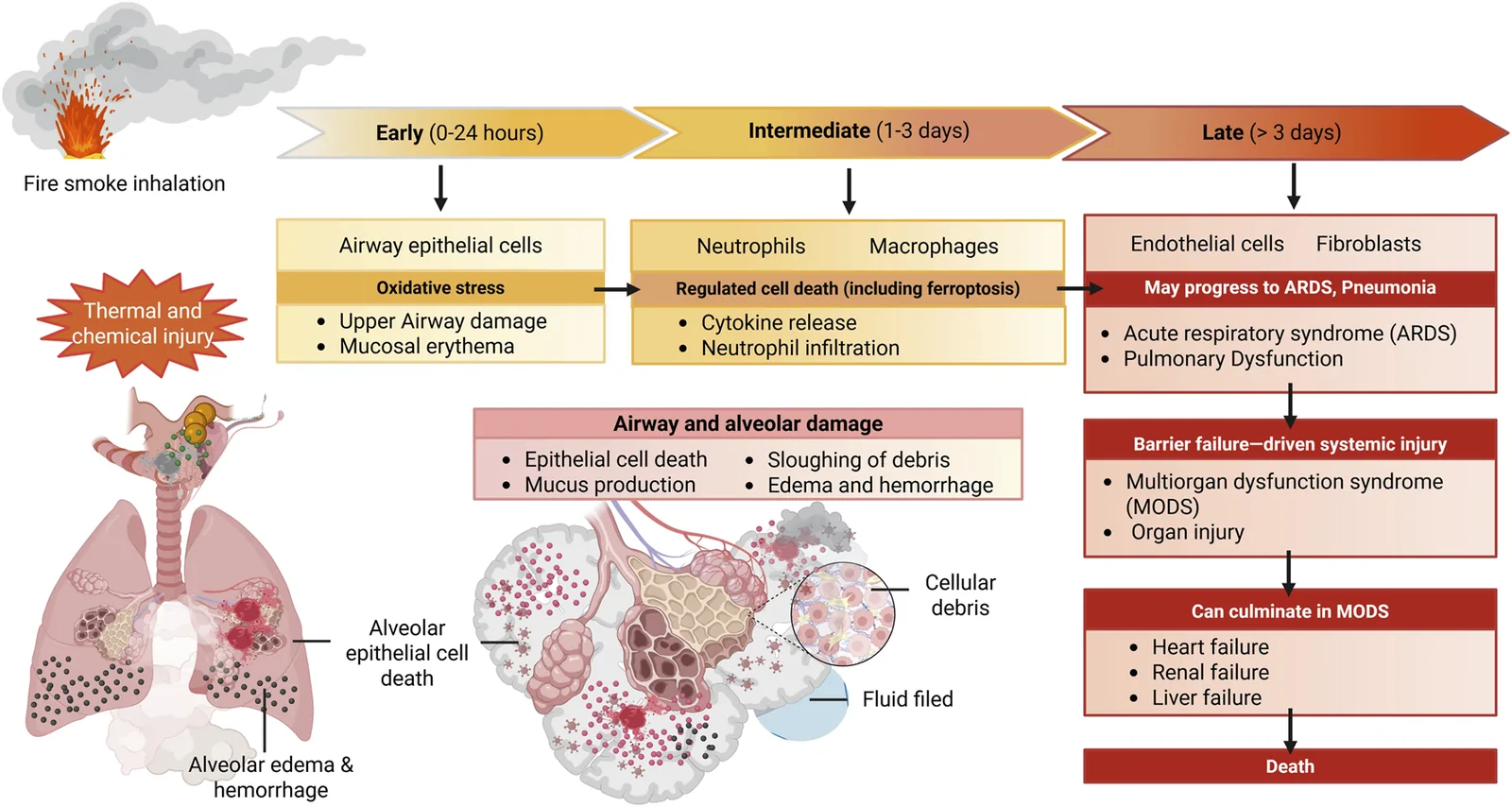
Pathological progression of SII. This schematic depicts the time-dependent pathophysiology of SII across early (0–24 h), intermediate (1–3 days), and late (>3 days) phases. Initial thermal and chemical insults to the airway induce epithelial damage, oxidative stress mediated by ROS, and mucus hypersecretion. These events trigger an inflammatory response characterized by cytokine release and neutrophil infiltration, leading to progressive airway and alveolar injury with epithelial cell death, debris accumulation, edema, and hemorrhage. Persistent alveolar–capillary barrier disruption results in impaired gas exchange, ARDS, and pneumonia. In severe cases, systemic inflammatory spread culminates multiple organ dysfunction syndrome (MODS) and death.

### 3.1 Toxic components-induced acute injury

SII represents one of the most severe complications in burn patients and remains a principal determinant of both fire-related mortality and post-admission deaths (Galeiras et al., 2020). Rather than resulting from a single insult, SII arises from concurrent exposure to thermal stress, particulate matter, and a heterogeneous mixture of toxic gases, which together initiate complex local and systemic pathophysiological cascades within the respiratory system and beyond (Gupta et al., 2018). Importantly, the anatomical distribution and severity of airway injury are not uniform but are shaped by the specific composition of inhaled smoke and the duration of exposure. Consequently, acute injury severity closely reflects both the concentration of toxic constituents and exposure time (Galeiras, 2021). Among these constituents, CO, HCN, and particulate matter have emerged as dominant drivers of early-phase SII, exerting synergistic effects that substantially worsen clinical outcomes (Lobo-Antuña et al., 2026).

CO is generated through incomplete combustion of organic materials and constitutes a colorless, odorless, yet highly lethal component of fire smoke (Wang and Zhang, 2024). Its toxicological significance lies primarily in its exceptionally high affinity for hemoglobin, approximately 200–250 times greater than that of oxygen, leading to the formation of COHb and a marked reduction in arterial oxygen-carrying capacity (Rose et al., 2017). This interaction not only limits oxygen delivery but also induces a leftward shift of the oxyhemoglobin dissociation curve, thereby exacerbating tissue hypoxia. Organs with high metabolic demand, particularly the brain and myocardium, are especially vulnerable. Beyond its effects on hemoglobin, CO directly impairs mitochondrial respiration by binding to cytochrome c oxidase, disrupting oxidative phosphorylation and cellular energy production (Afzal et al., 2025). Clinically, CO poisoning manifests along a spectrum ranging from mild neurological symptoms to coma and seizures, with early signs such as headache, dizziness, and nausea appearing at COHb levels of approximately 15%. Concentrations exceeding 30%–40% are typically associated with incapacitation or loss of consciousness (Permentier et al., 2017). Accurate diagnosis requires co-oximetry, as conventional pulse oximetry may yield falsely reassuring readings. Notably, CO exposure is linked not only to increased acute mortality but also to delayed neurological sequelae, a phenomenon attributed in part to lipid peroxidation and persistent oxidative injury within the central nervous system (Rose et al., 2017). Epidemiological data underscore this burden: more than half of fire-related injuries treated in emergency departments have been associated with anoxic injury, most commonly attributable to CO inhalation.

HCN represents another highly toxic gaseous component of fire smoke, predominantly released during the combustion of nitrogen-containing synthetic materials, including plastics, nylons, vinyls, and rubbers (Grabowska et al., 2012). Cyanide toxicity is mediated through potent inhibition of cytochrome oxidase, effectively arresting aerobic cellular respiration. As a result, tissues are rendered incapable of utilizing available oxygen, leading to histotoxic hypoxia, a rapid shift toward anaerobic metabolism, and the accumulation of lactic acid culminating in severe metabolic acidosis. Cardiovascular complications, including myocardial infarction, have been reported in fire victims with cyanide exposure. Clinically, cyanide poisoning presents with hypotension, respiratory distress, and markedly elevated plasma lactate levels (≥10 mmol/L), often developing within seconds of inhalation. At concentrations exceeding approximately 150 ppm, HCN can induce rapid incapacitation within minutes. Unlike CO intoxication, cyanide poisoning lacks a rapid, reliable bedside diagnostic test, and its clinical presentation often overlaps with that of CO exposure, complicating timely recognition and management (Habeb et al., 2025). Toxicological analyses of blood samples from fire victims have revealed cyanide concentrations within toxic ranges in nearly half of cases, with lethal levels detected in a significant subset, particularly in enclosed-space and vehicle-related fires. These findings highlight cyanide as a frequently underrecognized but critical contributor to fire-related morbidity and mortality.

Particulate matter constitutes a third major injurious component of fire smoke, encompassing solid and liquid particles of diverse size and chemical composition determined by the materials undergoing combustion (Laguerre and Gall, 2024). While larger particles are typically trapped within the upper airways, fine (PM2.5) and ultrafine (PM0.1) particles readily penetrate the distal airways and alveolar spaces (Schraufnagel, 2020). Their deposition provokes robust inflammatory responses, leading to epithelial injury, bronchospasm, mucus hypersecretion, and airflow obstruction. Beyond acute injury, sustained particulate exposure contributes to long-term pulmonary dysfunction in SII survivors. Experimental evidence indicates that particulate matter generated from the combustion of plastic-containing waste elicits disproportionately severe pulmonary inflammation and tissue damage compared with other combustion sources (Kim et al., 2021). Importantly, the pathological effects of particulate matter extend beyond the lungs. Fine particles have been implicated in systemic inflammatory activation, oxidative stress, endothelial dysfunction, and heightened susceptibility to infection (Riggs et al., 2020). Translocation of ultrafine particles into the circulation may precipitate cardiovascular events, while alternative pathways, such as olfactory nerve transport, allow access to the central nervous system, potentially contributing to vascular pathology and cognitive impairment (Schraufnagel, 2020). Chronic smoke exposure further predisposes individuals to obstructive airway diseases, including COPD and asthma (Xu K. et al., 2025). Collectively, CO, cyanide, and particulate matter act both independently and synergistically to drive the acute pathophysiology of SII. Their combined effects amplify tissue hypoxia, inflammatory injury, and systemic dysfunction, resulting in substantial morbidity and, in severe cases, increased mortality-particularly when recognition or intervention is delayed (Galeiras, 2021). A mechanistic understanding of these toxic constituents is therefore essential for improving both preventive strategies and targeted therapeutic approaches in fire-related inhalation injuries.

SII remains a severe and insufficiently treatable clinical condition. Its impact is particularly evident in burn medicine, where inhalation injury markedly increases mortality and can raise the risk of burn-related death by up to 20-fold. Despite this clinical burden, current management remains largely supportive, including airway protection, oxygen therapy, bronchoscopy-guided assessment, ventilatory support, and treatment of secondary complications. The absence of disease-modifying therapy does not reflect a lack of research activity; rather, it underscores a persistent translational gap between expanding preclinical knowledge and clinically actionable interventions. This gap is further complicated by the marked heterogeneity of SII across exposure conditions, clinical presentation, and biological response. Smoke is not a uniform injurious agent; each fire generates a distinct mixture of particulates, airway irritants, systemic toxins, and thermal stressors, which together shape the pattern and severity of tissue injury (Walker et al., 2015). At the patient level, disease trajectory is influenced by exposure intensity, smoke composition, inhaled temperature, ambient oxygen concentration, and the secondary systemic response triggered by inhalation injury (Herdy Guerra Avila et al., 2024). As a result, patients exposed to apparently similar fire environments may develop substantially different clinical courses. Even among patients with comparable exposure histories and bronchoscopic grades, the timing and extent of respiratory deterioration, pneumonia, and progression to ARDS may differ, suggesting that host-specific biological programs critically modulate disease evolution (Amarasekera et al., 2025).

Current pathological frameworks centered on oxidative stress, inflammation, and structural airway injury do not fully explain why patients with similar clinical injury grades experience divergent outcomes. Ferroptosis, an iron-dependent form of regulated cell death driven by lipid peroxidation, provides a mechanistically plausible but still insufficiently investigated explanation for this variability. Ferroptosis can be initiated through both extrinsic transporter-dependent pathways and intrinsic enzyme-regulated pathways, particularly when the balance between oxidant generation and antioxidant defense is disrupted by altered activity or expression of redox-active enzymes (Zhou et al., 2024c). In SII, patients with extensive hemoglobin degradation or microhemorrhage within the lung parenchyma may develop an expanded LIP, thereby increasing the likelihood of ferroptotic pathway activation compared with patients whose injury is largely confined to the airways. Similarly, profound GSH depletion caused by toxic combustion products may impair GPX4-dependent lipid peroxide detoxification, lower the threshold for ferroptotic cell death, and intensify epithelial and endothelial injury (Wen et al., 2024). On this basis, the present study considers ferroptosis not merely as one downstream manifestation of oxidative injury, but as a candidate biological determinant of interpatient variability in SII. By linking ferroptosis-related molecular mechanisms to clinically relevant injury patterns, this work seeks to refine the mechanistic framework of SII and support the development of targeted therapeutic strategies for patients who currently lack effective disease-modifying treatment (Holley et al., 2020).

### 3.2 Airway and alveolar structural damage and oxidative stress

Smoke inhalation brings about extensive morphological changes throughout the entire respiratory system, impacting both the airways and the alveoli. Most importantly, oxidative stress continues to be a well-known major factor in the damage done to the lungs by smoke (Ortiz-Quintero et al., 2022; Bezerra et al., 2023). The acute effects of smoke on airways include profound damage to cellular membranes within the trachea and bronchi. This type of injury manifests as an accompaniment of significant vascular leakage, submucosal edema, and swelling of the airway walls. This is often accompanied by necrotic tracheobronchitis, with necrosis and sloughing of the epithelial lining of the trachea and bronchi. Necrotic debris, sloughed with fibrin and inflammatory exudates, can form pseudo-membranes within the airways, which can obstruct airways, thus increase resistance and compromise the extent of ventilation. Edema, bronchospasm, excessive mucus, and pseudomembrane can turn into severe airway obstruction with the potential to become fatal. Inhalation of superheated air, especially steam, as the major cause of thermal damage, chiefly affects the upper airway, resulting in erythema, blistering, and ulceration. Cilia cell damage and morphologically altered mucus properties considerably impairs mucociliary clearance, one of the most important defense mechanisms against inhaled particles and pathogens. Increased chances of lower respiratory tract infections and death through airway plugging. Sustained oxidative stress may amplify ferroptosis-associated lipid peroxidation through dysregulation of the ACSL4/GPX4 axis, in which enhanced ACSL4-dependent lipid peroxidation and impaired GPX4-mediated antioxidant defense promote epithelial injury, inflammatory amplification, fibroblast activation, and collagen matrix deposition. This process provides a mechanistic link between acute ferroptotic epithelial damage and subsequent chronic airway remodeling (Doll et al., 2017; Yan X. et al., 2024). With the persistance of injury, inflammation along with improper repair processes leads to chronic remodeling of the airways, characterized by subepithelial fibrosis, hyperplasia of goblet cells, and hypertrophy of smooth muscles. Such characteristics of chronic airway remodeling finally lead to airflow limitation and dysfunction years down the line. Optical coherence tomography (OCT) studies have revealed that there is substantial airway thickening in animals exposed to smoke, especially in the lower compared to the upper trachea, indicating regional variations in the severity of injury (Miao et al., 2019). It’s fine structural arrangement is essential for gas exchange that could sustain severe smoke-injured damage (Budden et al., 2017). The epithelial damage due to smoke, inflammation, and oxidative stress mainly led to dysfunction in the alveolar barrier (Wang and Chao, 2025). For example, wildfire smoke has been known to induce oxidative stress that damages airway epithelial cells directly and can act as a trigger for the release of alarmins (Yu et al., 2025). The components of cigarette smoke, having many kinds of ROS and RNS, cause direct lung damage to alveolar epithelial cells, leading to cell death (apoptosis or necrosis), depending largely on the intensity and duration of exposure. This death is largely attributed to an increase in oxidative stress (Bezerra et al., 2023). Type II alveolar epithelial cells mainly contribute to normal alveolar architecture and its function, surfactant production, inflammation regulation, and repair processes. Damage to these types of cells would result in increased epithelial permeability, lowered surfactant production, and uncontrolled release of inflammatory cytokines and growth factors (Bi et al., 2025). Loss of alveolar structures, an important hallmark of pulmonary emphysema, is strongly associated with apoptosis of both alveolar epithelial and endothelial cells brought about by cigarette smoke (Ma et al., 2021). Furthermore, severe lung damage cites changes such as airway collapse, disrupted air-blood barriers, and apoptosis (Wang and Chao, 2025). Conversely, smoke inhalation from wood has been able to show that alveolar instability increases in relation to the exposure dose that, in turn, contributed to the lung injury through a two-hit mechanism: direct toxic injury as well as shear-stress injury due to instability of the alveoli (Upadhyay et al., 2024).

Oxidative stress, an overarching mechanism involved with powerful wide-ranging effects, is a pivotal event in airway injury and alveolar structural impairments in SII. Cigarette smoke contains more than 4,000 chemical constituents, including substantial amounts of ROS and RNS, such as superoxide anions, hydrogen peroxide, hydroxyl radicals, and peroxynitrite (Boukhenouna et al., 2018). Gaseous phase cigarette smoke contains a set of short-lived ROS, while its tar phase contains long-lived hydroquinones, which are responsible for creating persistent oxidative stress via redox cycling. Hydroquinones and hydrogen peroxide can permeate cells and enter the nucleus, thereby potentially causing oxidative DNA damage (Seo et al., 2023). Oxidative damage of increased alveolar epithelial cells may trigger cell arrest, detachment, lysis, and increased epithelial permeability (Zeng and Yan, 2025). Higher amounts of oxidative DNA, particularly 8-hydroxy-2′-deoxyguanosine (8-OHdG) have been reported in lung tissues following short periods of exposure to cigarette smoke, with increased damage in alveolar type II cells (Chen et al., 2015). Oxidative stress can also activate PARP, a DNA repair enzyme, leading to depletion of cellular levels of nicotinamide adenine dinucleotide (NAD^+^), impairment of ATP synthesis, and subsequent cell death. Chronic exposure to cigarette smoke also damages mitochondria in airway epithelial cells, thus promoting oxidative stress (Ma et al., 2021). The outcome seen here would be where oxidative stress essentially worsens lung injury upon smoke inhalation, and this would be as a result of the imbalance between pro-oxidant compounds and antioxidant defense mechanisms, especially where the activation of antioxidant system is insufficient (Albano et al., 2022). Passive smoke inhalation increases oxidative stress. Components in wildfire smoke also induce oxidative stress and systemic inflammation. Biomass burning aerosol research describes that oxidative stress and inflammation are two vital processes in the adverse health effects that increase airway resistance and damage alveoli. Wildfire smoke-derived PM2.5 causes lung injury by oxidative stress, local and systemic inflammation, compromise on airway epithelium, and aggravating the susceptibility to infection (Roscioli et al., 2018). Recent studies suggested that occupational exposures of firefighters lead to key toxic mechanisms that are known to contribute to cancer: oxidative stress, DNA damage, and chronic inflammation (Demers et al., 2022).

### 3.3 Inflammatory response and immune cell activation

SII triggers a complex, multilayered inflammatory response accompanied by broad activation and reprogramming of immune cell populations. This response not only drives the initial pulmonary insult but also contributes to delayed systemic complications and chronic sequelae (de Carvalho et al., 2016). The dynamic interplay between inhaled toxic constituents and host immune defenses ultimately determines injury severity, clinical trajectory, and patient outcomes following SII.

The respiratory tract mounts a rapid inflammatory reaction involving both innate and adaptive immune systems after smoke exposure (de Carvalho et al., 2016; Saaoud et al., 2023). A hallmark of acute SII is the swift and extensive recruitment of neutrophils into the airways and lung parenchyma. This influx is orchestrated by chemokines and cytokines released from injured epithelial cells and resident immune cells. Although neutrophils are essential for debris clearance and antimicrobial defense, their excessive activation promotes tissue injury through the release of proteases, myeloperoxidase, and large quantities of ROS. Numerous studies have demonstrated a direct correlation between neutrophil burden and the severity of inhalation injury, with higher neutrophil accumulation associated with worse lung damage (Dunn et al., 2018). In addition, smoke exposure induces a pronounced surge in pro-inflammatory cytokines and chemokines, detectable as early as 8 h post-inhalation (de Carvalho et al., 2016). Key mediators include IL-6, a central regulator of acute-phase responses and neutrophil mobilization, and TNF-α, a potent pro-inflammatory cytokine that also promotes apoptotic signaling (Leiphrakpam et al., 2021). Interleukin-8 (IL-8) serves as a dominant neutrophil chemoattractant and contributes to inflammasome activation. Collectively, these cytokines sustain granulocyte and macrophage recruitment and prolong immune cell retention within injured lung tissue (Cambier et al., 2023). Elevated concentrations of these mediators in bronchoalveolar lavage fluid (BALF) from SII patients are closely linked to airway hyperreactivity, bronchoconstriction, and pulmonary edema. In parallel, the complement cascade is rapidly activated during SII, with anaphylatoxins such as C3a and C5a acting as potent chemoattractants and amplifiers of inflammation (Detsika et al., 2024).

A transition from an early neutrophil-dominated innate response to T helper 17 cells (Th17)-skewed adaptive immunity represents a critical but incompletely defined component of SII immunopathology (Ritzmann et al., 2022). Rather than reflecting a passive temporal sequence, this transition appears to be actively orchestrated by danger-associated signals, cytokine remodeling, and the functional reprogramming of antigen-presenting cells (Thomas et al., 2023). During the acute phase of SII, neutrophils are rapidly recruited and activated, leading to NETosis and the release of neutrophil extracellular traps (NETs) composed of chromatin scaffolds, proteases, citrullinated histones, and DAMPs (Wang et al., 2024; Keir and Chalmers, 2022; Xuan et al., 2024). Excessive NET formation is increasingly recognized not only as an effector mechanism of tissue injury but also as an immunoregulatory platform that bridges innate and adaptive inflammation. In smoke-related airway diseases, exaggerated NET accumulation has been associated with greater disease severity and steroid resistance (Wang et al., 2025b).

DCs occupy a central position in this innate-to-adaptive immune shift. NET-derived DAMPs, particularly HMGB1, can activate pulmonary DCs and induce the production of IL-23, a cytokine essential for Th17 lineage stabilization (Zhang et al., 2014). Through this HMGB1-DC-IL-23 axis, NET-associated danger signals promote the differentiation of naïve CD4^+^ T cells toward IL-17-producing Th17 cells, thereby converting acute neutrophil-mediated injury into a more persistent adaptive inflammatory program. Consistent with this model, smoke-exposed experimental systems show enhanced DC activation and increased expression of Th17-polarizing cytokines, including IL-6 and IL-23 (Mengistu et al., 2026). The cytokine milieu further determines the magnitude and persistence of Th17 differentiation: IL-6, IL-1β, and TGF-β cooperate to activate STAT3 and induce RORγt, the master transcription factor governing Th17 lineage commitment (Sutanto et al., 2025). IL-21 amplifies this differentiation program, whereas IL-23 reinforces the stability and pathogenicity of established Th17 cells. In smoke-injured lungs, sustained IL-6 signaling may shift T-cell differentiation away from regulatory phenotypes and toward pathological Th17 expansion (Thomas et al., 2023).

Smoke constituents may further intensify this Th17-polarizing circuit through aryl hydrocarbon receptor (AhR)-dependent signaling, as polycyclic aromatic hydrocarbons (PAHs), abundant in inhaled smoke, act as AhR ligands capable of promoting Th17 differentiation (Talbot et al., 2018). Supporting this mechanism, AhR activation enhances IL-17 production, whereas disruption of IL-17 signaling attenuates smoke-induced lung injury. Inflammasome activity also contributes to this axis, as IL-1R1- and NLRP3-deficient models display reduced IL-17A expression (Le Rouzic et al., 2017). Once established, Th17 responses can sustain a self-amplifying inflammatory circuit: IL-17 induces neutrophil-recruiting chemokines, thereby perpetuating neutrophil influx, airway inflammation, and secondary tissue injury (Le Rouzic et al., 2017; Alcorn et al., 2010). This feed-forward loop provides a plausible mechanistic basis for persistent airway remodeling and steroid-refractory inflammation after smoke exposure. In parallel, remodeling of DC subsets, particularly the emergence of cDC2-dominant antigen-presenting networks, may further bias adaptive immunity toward Th17 polarization and reinforce immune disequilibrium in the injured lung (Mengistu et al., 2026). Collectively, these findings support a staged immunopathological model of SII in which neutrophil activation and NET release initiate DAMP-driven DC activation, followed by cytokine remodeling and sustained Th17-mediated pathology.

Smoke inhalation also disrupts the functional integrity of multiple immune cell populations, affecting both innate and adaptive immunity (Johnson et al., 2025). Resident AMs are central regulators of pulmonary immune homeostasis, orchestrating both the initiation and resolution of inflammation. Following smoke exposure, AMs undergo phenotypic activation and markedly increase the production of pro-inflammatory mediators (Lugg et al., 2022). Although their phagocytic capacity is transiently impaired in the early phase, it subsequently recovers, enabling the clearance of apoptotic neutrophils and cellular debris. However, sustained or dysregulated AM activation promotes chronic inflammation, airway remodeling, and fibrotic responses (Xie et al., 2025). Notably, bone marrow-derived mesenchymal stromal cells have been shown to attenuate SII by modulating AM polarization, highlighting a potential therapeutic strategy targeting macrophage plasticity (de Carvalho et al., 2016). Dendritic cells (DCs) provide a critical bridge between innate and adaptive immunity. Upon activation in the lung, DCs migrate to regional lymph nodes, where they prime naïve T lymphocytes and shape adaptive immune responses following smoke exposure. Mouse models of SII have demonstrated enhanced activation of bone marrow–derived DCs and subsequent T-cell proliferation. Concurrently, smoke inhalation reduces the numbers of lung-resident CD4^+^ and CD8^+^ T cells, natural killer cells, and B cells, thereby weakening local adaptive immunity and increasing susceptibility to secondary infections. In contrast, Th17 cells are preferentially skewed toward promoting persistent inflammation in smoke-associated lung diseases (Olson et al., 2026). Wildfire smoke exposure has additionally been shown to induce mixed eosinophilic and neutrophilic airway inflammation through AhR-dependent signaling in immune cells. Importantly, prospective analyses have shown that non-survivors of burn and SII exhibit markedly reduced levels of key immuno-modulators in BALF, including C5a, IL-1β, and IL-8, indicating that early pulmonary immune hyporesponsiveness is associated with increased mortality risk. Conversely, cigarette smoking has been consistently linked to increased inflammatory cell counts in BALF, particularly neutrophils, and focal inflammatory infiltrates within the lung (Davis et al., 2013). More recent studies further demonstrate that cigarette smoke exacerbates immune-inflammatory responses in chronic airway diseases, thereby sustaining pathological inflammation and contributing to disease progression, including COPD (Huber et al., 2023).

Oxidative stress and inflammation are tightly coupled upstream drivers of SII and establish a self-perpetuating cycle of tissue injury. Smoke constituents and activated immune cells generate excessive ROS, which act both as cytotoxic agents and signaling molecules that activate pro-inflammatory transcription factors such as NF-κB, thereby amplifying cytokine production and inflammatory signaling cascades (Guo et al., 2019). Oxidative stress further promotes inflammasome activation, facilitating the processing of pro-IL-1β into its mature form and triggering pyroptotic cell death (Li X. et al., 2025). In addition, smoke-induced mitochondrial dysfunction enhances the release of DAMPs, reinforcing immune activation and sustaining inflammation (Liu Y. et al., 2024). The inflammatory response elicited by SII often persists well beyond the acute phase, giving rise to systemic manifestations and long-term pulmonary dysfunction. Inflammation in the airways and peripheral circulation has been reported to persist for at least 6 months following inhalation injury, contributing to chronic airflow limitation and obstructive lung disease (Park et al., 2003). In severe cases, SII can precipitate systemic inflammatory response syndrome (SIRS), leading to multiple organ dysfunction syndrome (MODS), a major determinant of mortality. Beyond the lung, biomass smoke exposure has been shown to induce neuroinflammatory and neurodegenerative changes in preclinical models, highlighting the far-reaching consequences of smoke-induced immune dysregulation (Moshensky et al., 2022). Recent comprehensive reviews further emphasize that wildfire smoke exposure exerts profound and lasting effects on the human immune system, both acutely and chronically (Lei Y. et al., 2024). Collectively, these findings underscore inflammation and immune cell activation as central determinants of both local lung injury and systemic pathology following smoke inhalation.

## 4 Role of ferroptosis in SII

Ferroptosis, a metabolically regulated form of cell death driven by iron-dependent lipid peroxidation, has emerged as a central pathogenic mechanism in SII. Inhalation of combustion products disrupts pulmonary iron homeostasis through hemoglobin degradation and the subsequent release of redox-active iron, thereby amplifying lipid peroxidation and inflammatory signaling cascades within lung parenchyma. The convergence of iron overload, oxidative membrane damage, and inflammation not only precipitates extensive structural and functional lung injury but also positions ferroptosis as a nodal process intersecting with other cell death programs in smoke-exposed tissues (Yang et al., 2022). Elucidating these interdependent mechanisms provides a conceptual framework for understanding how ferroptosis orchestrates cellular demise in SII rather than acting as an isolated death pathway (Figure 4).

**FIGURE 4:**
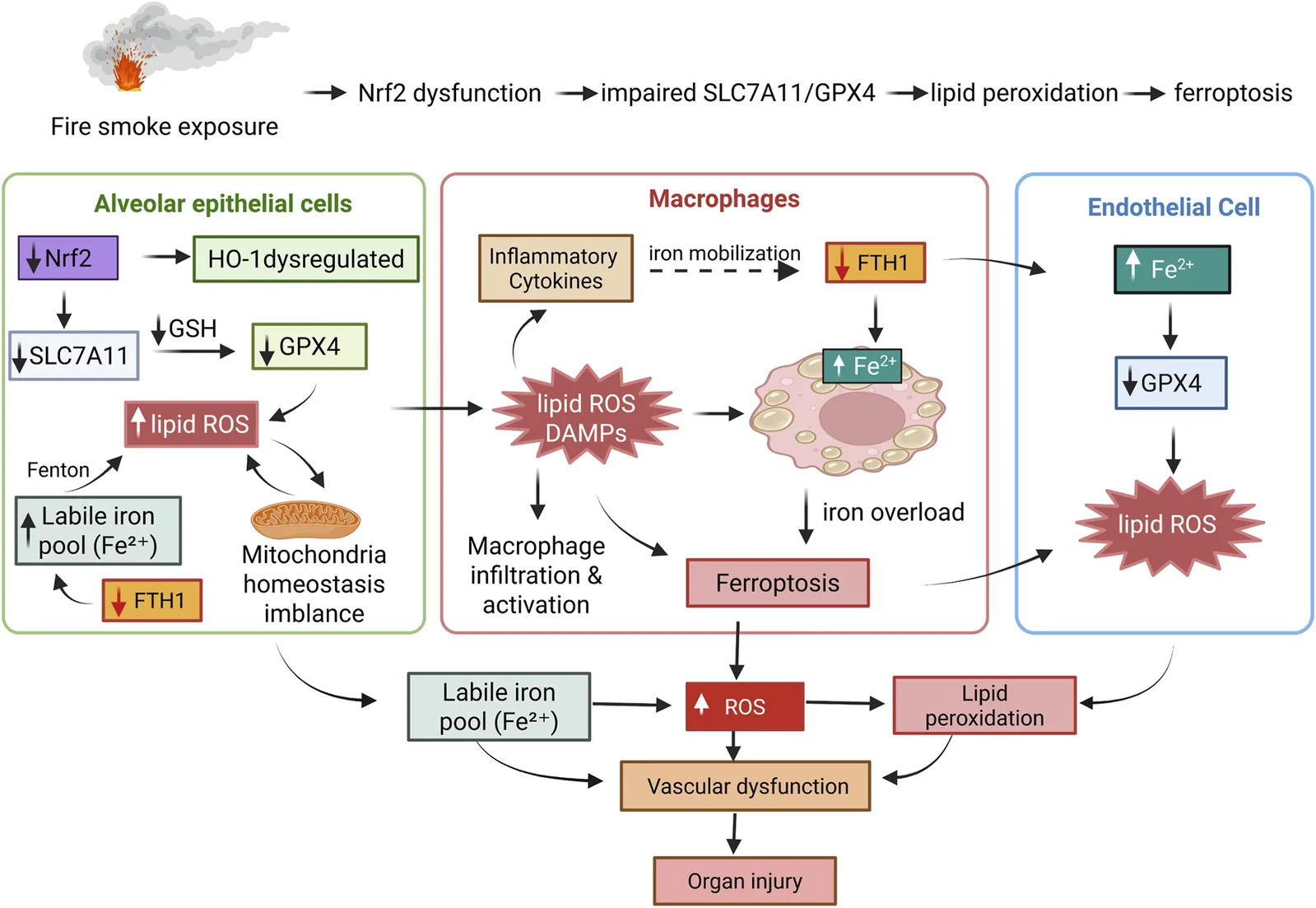
Mechanistic diagram linking ferroptosis and SII. Smoke inhalation induces oxidative stress, inflammation, and mitochondrial dysfunction, leading to ferroptosis in alveolar epithelial cells, macrophages, and endothelial cells. Disruption of the Nrf2-HO-1/SLC7A11-GPX4 axis, iron (Fe^2+^) accumulation, and excessive ROS promote lipid peroxidation. Macrophage-derived inflammatory cytokines and lipid ROS amplify this response, while endothelial iron dysregulation further exacerbates oxidative damage. The convergence of iron overload, ROS, and lipid peroxidation drives ferroptosis, resulting in vascular dysfunction and organ injury.

### 4.1 Hemoglobin breakdown induces iron accumulation

SII induces alveolar hemorrhage, leading to erythrocyte lysis and the release of hemoglobin into the alveolar space, thereby constituting a major source of iron overload in lung tissue. Disruption of the alveolar-capillary barrier permits erythroplasm infiltration, followed by secondary cellular lysis and liberation of intracellular constituents. Extracellular hemoglobin subsequently initiates a pathogenic cascade characterized by oxidative stress and inflammatory amplification, a process that can be mitigated by haptoglobin administration in experimental models (Irwin et al., 2015). Central to hemoglobin-derived iron handling is HO-1, an inducible stress-responsive enzyme that catalyzes the degradation of free heme into biliverdin, ferrous iron, and CO in a cytochrome P450 reductase-dependent manner. By detoxifying free heme released from destabilized hemoproteins, HO-1 limits heme-mediated cytotoxicity; however, the concomitant release of Fe^2+^ significantly expands the LIP within lung tissue (Li Y. et al., 2023). This pool of redox-active, non-protein-bound iron serves as a catalytic substrate for Fenton chemistry, thereby promoting the generation of highly ROS. In current clinical practice, SII is mainly managed with anti-inflammatory interventions, among which glucocorticoids such as methylprednisolone are commonly used to suppress proinflammatory cytokine production and attenuate neutrophil-mediated alveolar damage. However, glucocorticoid therapy primarily interferes with downstream inflammatory signaling rather than the upstream iron-releasing events that initiate oxidative injury. Mechanistically, glucocorticoid-receptor complexes inhibit transcriptional programs driven by NF-κB and AP-1, but they do not directly regulate HO-1 activity or the liberation of Fe^2+^ that expands the LIP (Song et al., 2019). This distinction is important because HO-1 occupies a context-dependent position in ferroptosis regulation. Under physiological or moderately induced conditions, HO-1 contributes to antioxidant defense and cytoprotection; however, excessive HO-1 activation may accelerate heme degradation, increase intracellular Fe^2+^ accumulation, intensify Fenton reaction-derived ROS generation, and promote GPX4 insufficiency, thereby driving membrane lipid peroxidation and ferroptotic cell death. Pharmacological modulation of HO-1 therefore represents a mechanistically distinct strategy from glucocorticoid-based anti-inflammatory therapy and warrants further investigation in SII (Ryter, 2022). Supporting this concept, cigarette smoke has been shown to disrupt pulmonary iron homeostasis and amplify oxidative stress, while excessive ferritinophagy-driven ferroptosis in lung epithelial cells has emerged as a potential therapeutic target beyond existing anti-inflammatory regimens (Li Y. et al., 2023). Experimental evidence further supports a causal link between dysregulated systemic iron homeostasis and pulmonary iron deposition. Hepcidin-deficient mice exhibit elevated circulating iron levels that correlate with increased pulmonary iron accumulation, and repeated smoke inhalation consistently results in iron deposition within lung tissue across animal models (Jiang et al., 2025). At the cellular level, *in vitro* studies suggest that smoke exposure alters iron handling in AMs, favoring intracellular iron sequestration and persistence (Lugg et al., 2022).

Excess iron accumulation exerts profound biological consequences by amplifying oxidative stress and sensitizing cells to ferroptosis. Free iron catalyzes hydrogen peroxide conversion into hydroxyl radicals, inflicting oxidative damage on membrane lipids, proteins, and nucleic acids—hallmark events that converge on ferroptotic cell death pathways (Berndt et al., 2024). Clinical and experimental observations reinforce this paradigm: iron overload induced by intravenous iron dextran alters hepatocellular morphology and elevates oxidative stress markers, while human studies associate iron excess with increased lipid peroxidation, protein carbonylation, and telomere attrition (Punchai et al., 2024). In the lung, iron accumulation arising from inhaled pollutants or cigarette smoke poses a particular hazard due to the catalytic role of iron in radical formation. Accordingly, pulmonary iron levels are tightly regulated, and sustained disruption of iron metabolism, manifested by iron-laden macrophages and iron-driven oxidative stress, is increasingly recognized as a pathological feature of chronic lung diseases, including IPF (Luo et al., 2025).

### 4.2 Role of lipid peroxidation and ROS in SII

Iron-catalyzed Fenton chemistry constitutes a pivotal molecular mechanism linking iron overload to lipid peroxidation in smoke-injured lung tissue. Fe^2+^ reacts with hydrogen peroxide (H_2_O_2_) to generate highly reactive hydroxyl radicals (•OH), which preferentially attack PUFA-enriched phospholipids within cellular membranes (Shen et al., 2025). Such oxidative assaults initiate chain reactions of lipid peroxidation, culminating in the accumulation of iron-dependent lipid hydroperoxides, a defining biochemical hallmark of ferroptosis. At the molecular level, ferroptosis is characterized by the failure of cellular antioxidant defenses to restrain lipid peroxidation. Iron-driven radical formation promotes GSH depletion and functional inactivation of GPX4, thereby removing a critical enzymatic barrier to lipid hydroperoxide detoxification (Ursini and Maiorino, 2020). The resulting unchecked propagation of lipid peroxyl radicals destabilizes membrane integrity and commits lung cells to ferroptotic death.

Experimental and clinical evidence consistently demonstrates elevated lipid peroxidation in smoke-induced lung injury. Malondialdehyde (MDA) and 4-HNE, canonical end products of lipid peroxidation, are markedly increased in lung tissues following smoke inhalation (Seo et al., 2024). Animal models of SII uniformly exhibit elevated pulmonary MDA levels, while *in vitro* studies confirm increased MDA and 4-HNE production after exposure to cigarette smoke extract (CSE) or other inhaled toxicants. Notably, CSE-induced lipid peroxidation in epithelial cells can be attenuated by ferroptosis inhibitors, underscoring a causal relationship between lipid oxidative damage and ferroptotic signaling. The synthetic radical-trapping antioxidant Fer-1 and the iron chelator DFO have demonstrated protective effects against cigarette smoke-induced oxidative injury by suppressing ferroptosis, limiting lipid peroxidation, and reducing ferroptosis-associated biomarkers in experimental models (Tang D. et al., 2021). These agents counter iron-dependent oxidative damage through mechanisms that are fundamentally distinct from glucocorticoid pharmacology. Glucocorticoids primarily repress proinflammatory gene transcription, thereby attenuating neutrophil-driven inflammation, superoxide generation, and secondary ROS accumulation; they also influence vascular and endothelial responses by modulating inflammatory mediators such as phospholipase A2, cyclooxygenase, and nitric oxide synthase (Thompson, 2003; Amratia et al., 2022). However, they neither directly scavenge lipid peroxyl radicals nor chelate catalytic iron. By contrast, DFO sequesters non-transferrin-bound iron and LIP components, including iron associated with transferrin-ferritin exchange, ferritin, and hemosiderin, thereby limiting Fenton chemistry and downstream lipid peroxidation (Delgado-Martín and Martínez-Ruiz, 2024). In parallel, Fer-1 interrupts ferroptotic propagation by trapping lipid peroxyl radicals and preserving membrane phospholipid integrity. Thus, Fer-1 and DFO should not be viewed as substitutes for anti-inflammatory therapy but as mechanistically complementary probes and potential adjunctive strategies for targeting the iron-lipid peroxidation axis in SII. Nevertheless, the clinical translation of Fer-1 remains constrained by unfavorable pharmacokinetic properties, metabolic instability, uncertain efficacy in humans, and insufficient long-term safety data, which has prompted the development of Fer-1 analogs with improved plasma stability (Chen Y. et al., 2024). Similarly, fine particulate matter (PM2.5) exposure has been shown to induce lipid peroxidation-dependent ferroptosis in models of lung injury. The balance between lipid peroxidation and antioxidant defense is tightly regulated by ACSL4 and GPX4. ACSL4 promotes ferroptosis by facilitating the esterification and membrane incorporation of PUFAs, thereby increasing the susceptibility of phospholipids to peroxidative damage (Jin et al., 2024). In contrast, GPX4 functions as a central anti-ferroptotic enzyme by reducing lipid hydroperoxides to non-toxic lipid alcohols. Reduced GPX4 expression has been reported in COPD animal models, further implicating impaired lipid peroxide detoxification in smoke-related lung pathology (Ruan et al., 2025).

Mitochondrial dysfunction further exacerbates oxidative stress and lipid peroxidation in smoke-injured lungs. Smoke exposure disrupts mitochondrial morphology, induces fragmentation, and enhances mitochondrial ROS production in airway smooth muscle cells and alveolar epithelial cells. Concurrently, reductions in superoxide dismutase (SOD) activity and intracellular GSH levels compromise endogenous antioxidant capacity, shifting the redox balance toward a pro-oxidant state (Aghapour et al., 2022). Excessive ROS readily react with PUFA-containing membrane lipids to form lipid radicals (L•), which propagate lipid peroxidation and generate lipid-derived ROS (L-ROS), thereby reinforcing ferroptotic signaling cascades (Mortensen et al., 2023). Collectively, these processes establish lipid peroxidation and ROS amplification as central executioners of ferroptosis in SII.

### 4.3 Mutual amplification between ferroptosis and inflammation

Ferroptosis and inflammation are tightly coupled biological processes that reciprocally reinforce one another during lung injury. Ferroptotic cell death promotes inflammatory activation through the release of DAMPs and lipid peroxidation-derived mediators, while inflammatory signaling, in turn, exacerbates iron dysregulation and oxidative stress, thereby accelerating ferroptotic injury (Chen et al., 2023). Among these mediators, HMGB1 has been identified as a prototypical DAMP released from ferroptotic cells, serving as a potent amplifier of inflammatory responses in lung tissue (Wen et al., 2019). Pro-inflammatory cytokines further potentiate ferroptosis by amplifying oxidative stress and perturbing iron homeostasis. Elevated levels of IL-6 and TNF-α, both implicated in SII, contribute to tissue oxidative damage and sensitize cells to ferroptotic death (Wang et al., 2025c). In human airway epithelial cells, smoke condensate-induced transcriptional reprogramming of oxidative stress pathways is exacerbated by IL-6, which promotes lipid peroxidation and disrupts iron metabolism in bronchial epithelial cells (Guo et al., 2019). Immune cell recruitment represents a critical node in the ferroptosis-inflammation amplification loop. Smoke inhalation promotes the rapid accumulation and activation of macrophages and neutrophils within lung tissue, intensifying local oxidative stress and inflammatory burden (Li M. et al., 2023). Although AMs normally sequester iron in inert forms through ferritin storage—a process enhanced in hepcidin-deficient states—CSE disrupts macrophage gene expression programs and impairs phagocytic function, thereby favoring pathological iron retention and redox imbalance. Activated neutrophils further exacerbate injury by generating large quantities of reactive ROS, amplifying lipid peroxidation in surrounding cells (Figure 5).

**FIGURE 5:**
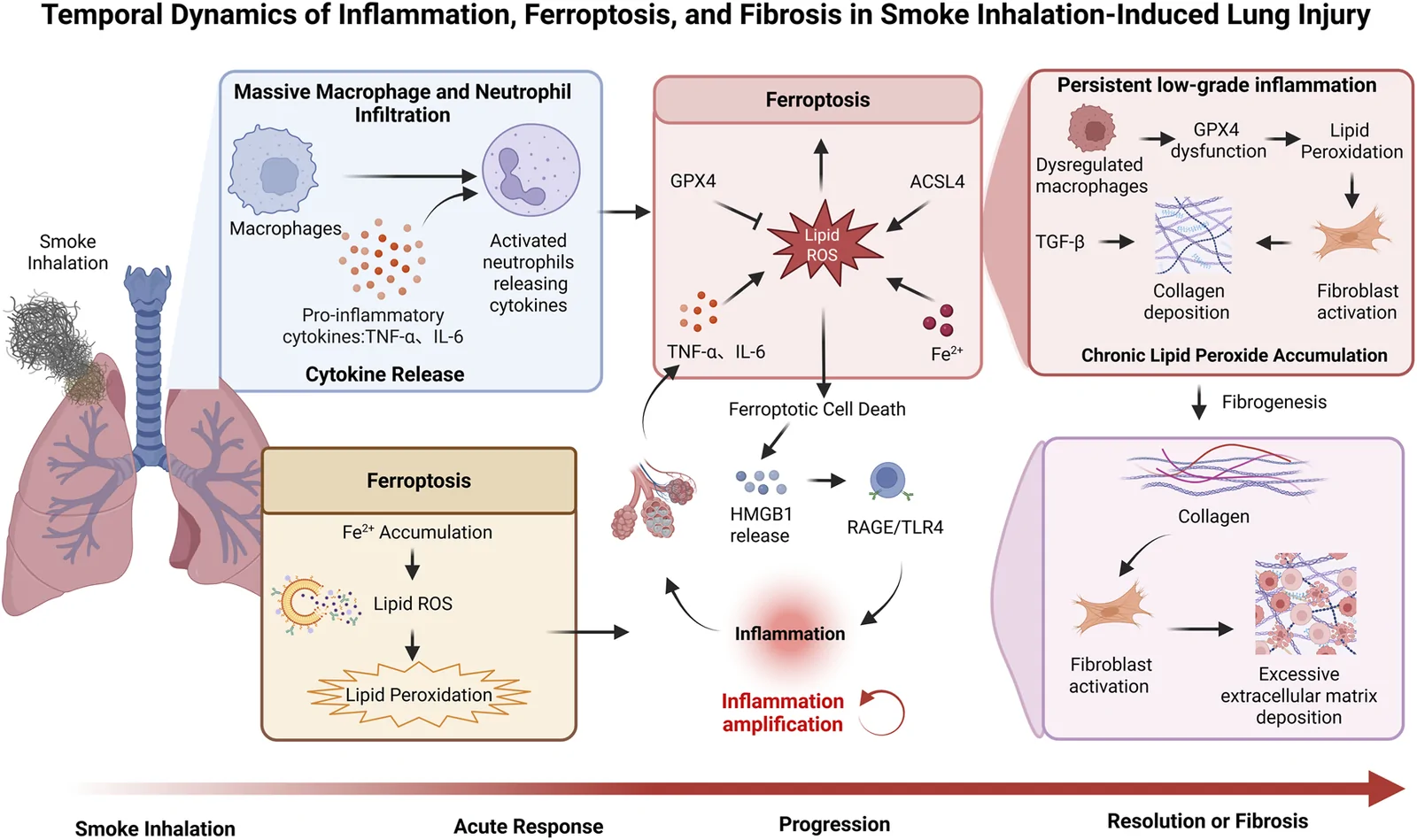
Temporal dynamics of inflammation, ferroptosis, and fibrosis in disease progression. During the early acute inflammatory phase, smoke exposure promotes extensive recruitment of macrophages and neutrophils and induces the release of pro-inflammatory cytokines, including TNF-α and IL-6. These inflammatory mediators aggravate oxidative stress, intracellular iron accumulation, and ACSL4-dependent lipid peroxidation, thereby initiating ferroptotic injury. Concurrent impairment of the GPX4-mediated antioxidant defense system further lowers the threshold for ferroptotic cell death. Ferroptotic cells release damage-associated molecular patterns (DAMPs), such as HMGB1, which activate RAGE/TLR4 signaling and reinforce inflammatory amplification, establishing a reciprocal feed-forward loop between inflammation and ferroptosis. As the injury evolves, persistent low-grade inflammation, sustained lipid peroxide accumulation, macrophage dysfunction, and TGF-β signaling collectively drive fibroblast activation, collagen deposition, extracellular matrix remodeling, and progressive pulmonary fibrosis. The lower timeline summarizes the pathological transition from smoke inhalation and acute inflammatory activation to ferroptosis-mediated tissue injury and subsequent fibrotic remodeling.

The regulatory complexity of this circuit is further shaped by endogenous pro-resolving and anti-inflammatory pathways that restrain ferroptotic propagation. Lipoxins are arachidonic acid-derived specialized pro-resolving lipid mediators that function as endogenous “stop signals” during inflammation. LXA4, acting through ALX/FPR2, has been shown to suppress cigarette smoke-induced lung injury by reducing lipid peroxidation and ferroptosis-associated markers through inhibition of p38 MAPK signaling (Li W. et al., 2025). In alveolar type II epithelial cells, LXA4/FPR2 signaling activates NRF2, increases GSH availability, decreases MDA accumulation, and limits ferroptotic injury. This protective pathway is receptor- and NRF2-dependent, as LXA4 reduces pulmonary dysfunction, ferroptosis, and inflammation in wild-type mice after lung ischemia-reperfusion injury, whereas these effects are lost in Fpr2^−/−^ or Nrf2^−/−^ mice. Beyond direct epithelial protection, LXA4/FPR2 signaling suppresses CXCL1 release, thereby limiting neutrophil trafficking from the circulation into the alveolar compartment (Kollareth et al., 2025). In COPD models, LXA4 also restores pulmonary function and attenuates emphysema and airway inflammation, accompanied by ALX/FPR2 upregulation and reduced p38 MAPK phosphorylation both *in vivo* and *in vitro*, and notably, the p38 MAPK inhibitor SB203580 similarly reverses CSE-induced inflammation and ferroptosis (Li J. et al., 2025).

IL-10 represents another endogenous brake on the ferroptosis-inflammation circuit. Produced mainly by regulatory T cells, macrophages, and dendritic cells, IL-10 exerts anti-inflammatory and anti-ferroptotic effects through multiple mechanisms. First, IL-10 can activate the IL-10R-STAT3 axis, leading to STAT3 phosphorylation and transcriptional support of GPX4, thereby reinforcing the enzymatic defense against phospholipid peroxide accumulation. Second, IL-10 suppresses TNF-α/NF-κB signaling and reduces ALOX15 expression, limiting lipoxygenase-driven lipid peroxidation. The relevance of this regulatory axis is supported by evidence that NRF2 and STAT3 cooperatively sustain SLC7A11 expression in intestinal ischemia-reperfusion-induced ALI, highlighting NRF2-STAT3 crosstalk as a potential anti-ferroptotic mechanism in inflammatory lung injury (Qiang et al., 2020). In the context of SII, simultaneous impairment of IL-10 production and GPX4 activity may weaken this endogenous protective network, thereby permitting unchecked amplification of ferroptosis and inflammation.

Additional specialized pro-resolving mediators may also participate in ferroptosis control during the resolution phase of lung injury. Protectin conjugates in tissue regeneration 1 (PCTR1), an endogenous lipid mediator involved in tissue repair, protects against lipopolysaccharide (LPS)-induced ALI and multiorgan damage in a manner functionally comparable to the ferroptosis inhibitor ferrostatin-1. Mechanistically, PCTR1 preserves mitochondrial ultrastructure and suppresses ferroptosis through activation of the ALX/PKA/CREB signaling cascade (Lv et al., 2023). These findings suggest that ferroptosis in the injured lung is not simply a terminal oxidative death program, but a dynamically regulated process controlled by the balance between pro-inflammatory drivers and pro-resolving counter-regulatory signals. In smoke-exposed lung tissue, the magnitude and duration of ferroptotic injury are likely determined by the relative dominance of IL-6, TNF-α, HMGB1, neutrophil-derived ROS, LXA4, IL-10, and PCTR1. Therapeutically, shifting this balance away from inflammatory iron-lipid peroxidation and toward endogenous resolution may provide a rational strategy for interrupting the ferroptosis-inflammation circuit in SII.

### 4.4 Ferroptosis-associated multimodal cell death in SII

Multiple forms of regulated cell death are activated in the smoke-injured lung, but their pathological significance in SII is best understood in relation to cell-type-specific vulnerability and the evolving inflammatory microenvironment. Smoke exposure imposes a combined burden of oxidative stress, toxic particulates, hypoxia-related injury, iron dyshomeostasis, and mitochondrial dysfunction. These insults do not trigger a single death pathway in isolation; rather, they create a tissue context in which ferroptosis, apoptosis, pyroptosis, and necrotic-like membrane injury may coexist or occur sequentially. Increased caspase-3 activation in smoke-exposed lung tissue supports the involvement of apoptosis in epithelial and endothelial cell loss, whereas ferroptosis proceeds independently of caspase activity and is characterized by iron-dependent lipid peroxidation and distinct ultrastructural changes, indicating that it represents a non-redundant contributor to SII pathology (Jin et al., 2026). Therefore, in SII, the key question is not whether ferroptosis replaces apoptosis or inflammatory cell death, but how ferroptotic lipid damage cooperates with these pathways to amplify epithelial injury, inflammatory recruitment, and barrier dysfunction.

The epithelial compartment is likely the principal site at which ferroptosis-associated cell death interactions shape SII progression. Bronchial and alveolar epithelial cells are directly exposed to smoke-derived particulates, aldehydes, CO, cyanide, and other combustion products, making them particularly susceptible to oxidative membrane damage. Cigarette smoke disrupts iron homeostasis and enhances oxidative stress, while excessive ferritinophagy further increases labile iron availability and promotes ferroptotic death in lung epithelial cells (Yoshida et al., 2019). This epithelial vulnerability has direct pathological implications. Ferroptosis in airway epithelial cells may contribute to epithelial denudation, airway inflammation, and remodeling, whereas ferroptosis in alveolar epithelial cells may impair alveolar integrity, reduce gas-exchange capacity, and promote emphysema-like or fibrotic remodeling. In this setting, apoptosis may account for part of the epithelial cell loss, but ferroptosis adds a distinct lipid peroxidation-driven injury module that can further compromise epithelial barrier function and propagate damage to neighboring cells.

AT2 cells appear to be a particularly important ferroptosis-sensitive population in smoke-related lung injury. As progenitor-like epithelial cells responsible for surfactant production and alveolar repair, excessive AT2 cell loss can impair epithelial regeneration and promote persistent alveolar dysfunction. Smoke exposure recruits macrophages, neutrophils, and lymphocytes into small airways and lung parenchyma, generating a cytokine- and lipid mediator-rich microenvironment that further sensitizes epithelial cells to ferroptosis. Monocyte-derived macrophages can release leukotriene B4 (LTB4), which induces ACSL4 expression in epithelial cells and promotes ferroptotic injury in AT2 cells (Yang et al., 2022). Consistently, persistent accumulation of monocyte-derived macrophages has been associated with ALOX5 overexpression, LTB4 metabolite accumulation, ACSL4 induction, and subsequent epithelial ferroptosis, supporting a macrophage-LTB4-ACSL4 axis as a key amplifier of AT2 cell injury in smoke-related lung disease (Günes Günsel et al., 2022). This mechanism links inflammatory cell recruitment to ferroptotic epithelial loss and provides a clearer explanation for how acute inflammation may evolve into defective repair and structural remodeling after SII.

Macrophages are not only upstream amplifiers of epithelial ferroptosis but also direct targets of ferroptosis-related injury. After smoke exposure, inhaled particulates deposit in the alveolar space and are taken up by AM’s, exposing them to oxidative and iron-related stress. Experimental models show ferroptosis-associated changes in macrophages, including decreased SOD activity and reduced GSH levels (Zheng et al., 2022). In addition, excessive erythrophagocytosis can enhance macrophage iron recycling, increase ROS production, and promote lipid peroxidation; these effects can be attenuated by ferrostatin-1 *in vitro* (Youssef et al., 2018). In SII, this process may be especially relevant because vascular leakage, epithelial disruption, and hemorrhagic components can increase heme and iron exposure in the alveolar microenvironment. Ferroptotic or dysfunctional macrophages may then fail to resolve inflammation efficiently, while damaged macrophages can also release inflammatory mediators and damage-associated signals that intensify epithelial stress. Thus, ferroptosis in macrophages may contribute to both impaired inflammatory resolution and secondary epithelial injury.

Pyroptosis provides another inflammatory layer within the SII cell death landscape. Although the general ferroptosis-pyroptosis crosstalk has been discussed above, its relevance to SII lies primarily in inflammatory amplification. Ferroptotic epithelial cells may release oxidized phospholipids, HMGB1, mitochondrial products, and other danger-associated molecular patterns that activate inflammasome signaling in neighboring AM’s. This can promote caspase-1 activation, gasdermin D cleavage, and IL-1β/IL-18 maturation, thereby converting local epithelial lipid peroxidation into a broader inflammatory response. Pyroptosis has been recognized as a critical pathogenic mechanism in ALI, particularly in sepsis-associated contexts where dysregulated interactions among pyroptosis, autophagy, and ferroptosis exacerbate inflammatory tissue damage (Shen et al., 2024). In SII, a similar process may operate in the smoke-injured alveolar niche: ferroptotic epithelial damage provides inflammatory triggers, while pyroptotic macrophage responses amplify cytokine release, neutrophil recruitment, and tissue injury.

Vascular and stromal compartments may further extend the impact of ferroptosis-associated multimodal cell death. Pulmonary endothelial cells are exposed to circulating inflammatory mediators, oxidative stress, and redox-active toxicants after smoke inhalation. Redox imbalance in the pulmonary vascular compartment can damage membrane lipids and sensitize endothelial cells to ferroptosis, while PM2.5 exposure further increases endothelial ferroptotic susceptibility (Wang and Tang, 2019). Endothelial injury may aggravate alveolar-capillary barrier leakage, edema formation, and inflammatory cell influx, thereby reinforcing the injurious microenvironment. Fibroblasts and other mesenchymal cells may also be influenced indirectly by ferroptotic epithelial damage and macrophage-derived mediators, contributing to extracellular matrix remodeling and fibrotic repair responses. Together, these observations suggest that ferroptosis in SII is not confined to a single cell type but participates in a multicellular injury network involving epithelial cells, macrophages, endothelial cells, fibroblasts, and recruited immune populations (Yan M. et al., 2024).

Necrotic-like membrane injury and regulated necrosis may represent late or severe manifestations of this multimodal death landscape. In heavily injured lung regions, persistent oxidative stress, mitochondrial dysfunction, and membrane lipid peroxidation can compromise plasma membrane integrity, producing injury patterns that overlap morphologically with necrotic cell death (Jiang et al., 2021; Tang X. et al., 2021). Rather than treating necrosis as an isolated endpoint, it may be more appropriate in SII to view necrotic-like damage as the consequence of unresolved oxidative and inflammatory stress, in which ferroptotic lipid peroxidation, pyroptotic pore formation, and other membrane-disruptive processes collectively contribute to epithelial and endothelial barrier failure (Samson et al., 2020; Xia et al., 2019). This perspective helps explain why severe SII often presents with diffuse alveolar damage, edema, inflammatory exudation, and progressive respiratory dysfunction (Zhang et al., 2021).

Overall, ferroptosis should be interpreted as a central but not solitary component of the SII cell death program. Its major contribution lies in connecting smoke-induced iron dysregulation and lipid peroxidation with epithelial loss, macrophage dysfunction, inflammasome activation, endothelial barrier injury, and aberrant tissue remodeling. This has important therapeutic implications. Selective inhibition of one death pathway may be insufficient if ferroptosis, apoptosis, pyroptosis, and necrotic-like injury are activated in parallel within the smoke-injured lung. A more rational strategy may require targeting shared upstream drivers, such as oxidative stress, iron overload, ferritinophagy, mitochondrial dysfunction, and inflammatory lipid mediators, while preserving epithelial repair capacity. Such an approach may better limit acute tissue injury and reduce the transition from early inflammation to persistent remodeling in SII.

## 5 Experimental evidence of ferroptosis and ferroptosis-related biomarkers

### 5.1 Evidence of ferroptosis

Ferroptosis has emerged as a non-canonical yet mechanistically distinct mode of regulated cell death that contributes substantially to the pathophysiology of SII. Accumulating experimental evidence indicates that iron-dependent lipid peroxidation constitutes a central driver of smoke-induced pulmonary damage (Yang et al., 2025). Accordingly, recent investigations have focused on delineating the molecular mechanisms underlying smoke-triggered ferroptosis and validating its pathological relevance across experimental systems. *In vitro* studies consistently demonstrate that cigarette smoke condensate (CSC) or CSE induces ferroptotic cell death in human bronchial epithelial cells (Zi et al., 2023). Mechanistically, CSE exposure suppresses MFG-E8 expression, disrupts iron homeostasis, and promotes lipid peroxidation, thereby precipitating ferroptosis. Beyond conventional culture systems, advanced exposure platforms including real-time aerosol delivery systems and biomimetic lung-on-chip models have further substantiated ferroptosis as a dominant epithelial injury pathway following cigarette smoke aerosol exposure (Cui et al., 2023). The induction of ferroptosis exhibits both dose- and time-dependence. In BEAS-2B cells and primary human bronchial epithelial cells, CSE exposure elicits transient increases in ferroptotic markers, followed by partial recovery associated with NCOA4 upregulation, suggesting dynamic regulation of ferritinophagy during early injury phases. These cellular observations are corroborated by animal models of SII in mice and rats, which recapitulate hallmark features of human pathology, including pulmonary edema, neutrophil infiltration, elevated inflammatory cytokines, and increased proteolytic activity in BALF (Jin et al., 2026). Notably, interspecies differences in immune responsiveness underscore the importance of model selection when extrapolating ferroptosis-related findings. Collectively, these experimental paradigms, spanning cell-based systems, engineered exposure platforms, and *in vivo* models, provide convergent evidence that ferroptosis is a biologically relevant and reproducible mode of lung epithelial injury induced by smoke exposure.

### 5.2 Ferroptosis-related biomarkers

#### 5.2.1 GPX4 expression or activity

GPX4 is a central antioxidant enzyme responsible for detoxifying phospholipid hydroperoxides and is widely regarded as a molecular hallmark of ferroptosis. In bronchial epithelial cells exposed to CSE, GPX4 expression and activity are markedly reduced, concomitant with increased lipid peroxidation and cell death (Chen et al., 2025). Conversely, pharmacological or phytochemical interventions that restore GPX4 expression, such as Ginkgo biloba extract, significantly attenuate ferroptotic injury *in vitro*. Beyond experimental models, GPX4 has attracted interest as a clinically relevant biomarker. Reduced GPX4 levels correlate with disease severity in COPD models and predict adverse outcomes in patients with sepsis, underscoring its potential utility in prognostic stratification (She et al., 2025).

#### 5.2.2 MDA and other lipid peroxidation products

MDA is a widely recognized biomarker of lipid peroxidation and a critical indicator of ferroptotic processes. In bronchial endothelial cells exposed to CSE, pretreatment with ferroptosis inhibitors such as Fer-1 significantly reduced ROS accumulation and downstream inflammatory mediator expression, indicating decreased lipid peroxidation (Lian et al., 2021). Similarly, Ginkgo biloba extract attenuated CSE-induced ROS generation and MDA formation in human bronchial epithelial cells (Xu A. et al., 2025). In animal models, knockdown of SHP2 in LPS-induced ALI decreased MDA production, concomitant with diminished ferroptotic activity. Elevated MDA levels were consistently observed in smoke-exposed animals and humans, linking cigarette smoke exposure to lipid peroxidation-mediated cellular injury. Therapeutic interventions that reduce MDA levels, including saline enriched with hydrogen, may alleviate oxidative stress and ferroptotic damage (Wang et al., 2024).

#### 5.2.3 Ferritin, LIP, and other iron metabolism markers

Iron accumulation, particularly within the LIP, is a principal driver of ferroptosis through Fenton chemistry and subsequent ROS generation. Ferritin, as the primary iron-storage protein, reflects cellular iron homeostasis and undergoes dynamic regulation in response to oxidative stress. CSE exposure elevates FTH1 mRNA in bronchial endothelial cells, while interventions such as Ginkgo biloba extract enhance GSH production and ferritin expression, mitigating ferroptosis in human bronchial epithelial cells (Mercel et al., 2020). In COPD animal models, ferritin deposition is significantly increased following chronic cigarette smoke exposure (Yoshida et al., 2019). Clinical studies further demonstrate elevated serum ferritin levels in smokers compared with non-smokers, suggesting systemic perturbation of iron homeostasis due to tobacco exposure (Lee et al., 2016). Recent findings indicate that CD63 regulates the secretion of iron-loaded ferritin via extracellular vesicles, linking intracellular iron metabolism with inflammatory signaling (Yanatori et al., 2021). Dysregulated iron metabolism contributes not only to ferroptotic cell death but also to secondary inflammatory cascades and organ injury.

#### 5.2.4 ACSL4 and related enzymes that influence lipid composition

ACSL4, an acyl-CoA synthetase, preferentially incorporates PUFA into phospholipids, thereby sensitizing membranes to peroxidation and facilitating ferroptosis. In bronchial endothelial cells treated with CSE, ACSL4 mRNA is upregulated, consistent with enhanced lipid peroxidation (Wang and Xia, 2023). Conversely, phytochemical interventions such as Ginkgo biloba extract downregulate ACSL4 expression and mitigate ferroptosis in human bronchial epithelial cells. In LPS-induced ALI models, SHP2 knockdown confers protection by suppressing ACSL4, whereas its overexpression exacerbates ferroptotic injury (Xu A. et al., 2025). Clinical correlations further indicate that elevated ACSL4 expression is associated with worse prognosis in conditions such as sepsis or cancer, highlighting its potential as a predictive biomarker (Zeng et al., 2025). Collectively, ACSL4 represents a mechanistic nexus linking lipid metabolism and iron-dependent cell death, underscoring its value as both a therapeutic and diagnostic target in smoke-induced pulmonary injury.

## 6 Ferroptosis-targeted interventions and candidate therapeutic modalities

Ferroptosis, an iron-dependent form of regulated cell death driven by lipid peroxidation, plays a pivotal role in the pathogenesis of smoke inhalation-induced lung injuries (Yang et al., 2025). Consequently, modulating ferroptosis represents a promising therapeutic avenue. Contemporary research has explored multiple intervention strategies, each characterized by distinct mechanisms, experimental evidence, and translational challenges.

### 6.1 Experimental ferroptosis inhibitors

#### 6.1.1 Ferroptosis-specific inhibitors: Fer-1 and Lip-1

Classical ferroptosis inhibitors, particularly Fer-1 and Lip-1, primarily suppress ferroptotic cell death by interrupting lipid radical propagation and limiting phospholipid peroxide accumulation (Scarpellini et al., 2023). As radical-trapping antioxidants, these compounds exhibit greater efficiency than α-tocopherol within phospholipid bilayers. Fer-1 has been reported to regulate the GPX4/ACSL3/ACSL4 axis and prevent lipid hydroperoxide accumulation, whereas Lip-1 attenuates mitochondrial ROS generation partly through modulation of VDAC1 and GPX4 (Zhang L. et al., 2024). In smoke-related models, Fer-1 reduced ferroptotic injury in human bronchial epithelial cells exposed to CSE (Tang X. et al., 2021). Beyond pulmonary systems, Fer-1 has shown protective effects in neonatal hypoxic-ischemic brain injury, while Lip-1 improved lung function and reduced inflammation in cold ischemia-reperfusion and radiation-induced lung injury models (Zhang H. et al., 2025). These findings support the biological feasibility of ferroptosis inhibition, although most evidence remains extrapolated from non-SII injury contexts.

A major barrier to the *in vivo* application of Fer-1 is its unfavorable pharmacokinetic profile. Despite potent *in vitro* activity, Fer-1 exhibits limited plasma stability, rapid metabolic degradation, and suboptimal systemic exposure. UAMC-3203, a second-generation Fer-1 analogue, was developed to overcome these limitations and demonstrates improved solubility, metabolic stability, and ferroptosis-inhibitory potency (Devisscher et al., 2018). Compared with Fer-1, UAMC-3203 shows markedly prolonged stability in human microsomes, stronger *in vitro* activity, and sustained plasma recovery after systemic administration. It also improved cardiac function after resuscitation in a rat model of cardiac arrest without overt toxicity after chronic administration. Nevertheless, its oral bioavailability remains limited, prompting the development of next-generation oxazole-based radical-trapping antioxidants with higher bioavailability and reduced clearance. These pharmacokinetic refinements are particularly relevant for lung injury, where rapid and sustained drug exposure at the epithelial surface may be more important than high systemic concentrations.

#### 6.1.2 Pulmonary delivery platforms and nucleic acid-based modulation

For SII, the route of delivery is not merely a formulation issue but a determinant of therapeutic plausibility. Inhaled administration is mechanistically attractive because it can concentrate anti-ferroptotic agents in the injured airway and alveolar compartments while minimizing systemic exposure. Pulmonary delivery bypasses hepatic first-pass metabolism and may achieve rapid local uptake, although mucociliary clearance, airway obstruction, edema, and oxidative degradation in the injured lung remain important barriers. Liposomal encapsulation may partly overcome these limitations by improving epithelial uptake, prolonging local retention, and enabling sustained release. Fer-1-containing liposomal formulations have restored GPX4 expression and reduced oxidative damage in several preclinical injury models. On pharmacological grounds, inhaled liposomal UAMC-3203 or newer oxazole-based radical-trapping antioxidants may represent a rational formulation concept for SII-specific testing, but this remains an inferential proposition. SII-specific pharmacokinetic studies, dose-response experiments, and therapeutic-window analyses are still required before these agents can be considered translational candidates (Mercel et al., 2020). Nanoparticle-based delivery platforms further expand the therapeutic possibilities of ferroptosis modulation. Lipid nanoparticles could theoretically deliver GPX4 mRNA, silence ACSL4, or modulate other ferroptosis-regulatory nodes within epithelial or macrophage populations. Such approaches may offer cell-selective intervention, but they remain at an early preclinical stage. Human pharmacokinetic data, pulmonary bioavailability profiles, and safety assessments for Fer-1, UAMC-3203, liposomal formulations, and nucleic acid-based ferroptosis therapies are currently lacking in SII-specific models.

### 6.2 Clinically repurposable anti-ferroptotic agents

#### 6.2.1 Iron chelators: deferoxamine and deferiprone

Iron chelators mitigate ferroptosis by reducing LIP, thereby limiting Fenton reaction-mediated ROS generation. DFO and deferiprone are clinically established chelators that enhance iron excretion and reduce tissue iron deposition (Abdukarimov et al., 2025). Novel deferric amine compounds demonstrate superior iron-binding efficiency and more potent inhibition of iron-triggered ferroptosis. In smoke and oxidative injury contexts, DFO attenuated ferroptosis and suppressed C-X-C motif chemokine ligand 8 (CXCL8) production in human nasal epithelial cells exposed to cigarette smoke (Pan et al., 2025). Curcumin, a natural antioxidant, also chelates iron and alleviates iron-mediated cellular damage (Lei L. et al., 2024). In hyperoxic lung injury models, increased iron accumulation coincided with alterations in ferroptosis-related proteins, which DFO reversed *in vitro* (Zhang Y. et al., 2025).

Among clinically available anti-ferroptotic strategies, DFO has unusually direct relevance to SII. In ovine smoke inhalation studies, aerosolized DFO-pentastarch reduced airway injury and lung dysfunction, whereas aerosolized DFO alone was less effective. These findings suggest that the therapeutic effect of DFO in SII depends not only on iron-binding capacity but also on airway retention and local bioavailability (Enkhbaatar et al., 2016; Lin et al., 2009). Colloid complexation appears to improve mucosal residence time and thereby enhances the ability of DFO to neutralize free iron released into smoke-injured airways. This point has important translational implications: for SII, aerosolized DFO-colloid complexes may provide a rational means of achieving local anti-ferroptotic activity while avoiding toxicity associated with high-dose systemic infusion.

Safety remains a central limitation. Systemic DFO at high doses has been associated with neurotoxicity and pulmonary toxicity, and clinical testing of high-dose systemic DFO in intracranial hemorrhage raised concern because of increased ARDS events (Selim et al., 2019). This is particularly relevant for SII patients, who already have a high baseline risk of ARDS, infection, hypoxemia, and hemodynamic instability. Deferiprone offers an alternative because it is orally available and membrane-permeable, allowing depletion of intracellular LIP (Skouta et al., 2014). However, its risk of agranulocytosis requires strict hematological monitoring, which complicates its use in critically ill burn or inhalation-injury populations. Thus, iron chelation remains one of the most clinically actionable anti-ferroptotic strategies, but its translation in SII will depend on local delivery, dose control, and rigorous safety monitoring.

#### 6.2.2 Antioxidants and redox-modulating agents: NAC, vitamin E, and melatonin

Antioxidants such as N-acetylcysteine (NAC), vitamin E, and melatonin confer protection by counteracting oxidative stress and ferroptotic signaling. NAC has been shown to attenuate ferroptosis-mediated lung injury in ischemia/reperfusion models and to synergize with statins in reducing inflammation (Zhou et al., 2024a). Vitamin E protects neurons from radiation-induced ferroptosis and promotes cell survival in germ cells, while melatonin inhibits ferroptosis via KEAP1/NRF2/PTGS2 and ATM/p53 pathways, with additional benefits in pulmonary disorders and cardiovascular protection (Yang et al., 2022; Ren et al., 2023). Combined use of antioxidants with ferroptosis-specific inhibitors may produce synergistic protection by partially chelating iron and reducing the necessary antioxidant dose.

#### 6.2.3 Dipyridamole (DIPY) and lipid peroxidation-suppressive repurposing

DIPY represents a clinically repurposable lipid peroxidation suppressor rather than a conventional anti-inflammatory agent. This approved antiplatelet drug has recently been shown to inhibit ferroptosis by scavenging lipid peroxyl radicals and interfering with ACSL4-dependent membrane lipid remodeling (Zhuang et al., 2025). In preclinical ARDS models, DIPY reduced lipid peroxidation and preserved alveolar barrier integrity, supporting its potential relevance to SII. Its established safety profile, oral bioavailability, and availability of intravenous formulations strengthen its translational appeal. However, its antiplatelet activity requires careful evaluation in burn patients, particularly those with bleeding risk, coagulopathy, or a need for invasive procedures. Thus, DIPY may be most appropriately considered as a repurposing candidate for biomarker-selected patients with pronounced lipid peroxidation rather than as a broadly applicable therapy for all SII cases.

#### 6.2.4 Combined anti-inflammatory and anti-ferroptotic strategies

Ferroptosis and inflammation form a reciprocal injury circuit in SII. Ferroptotic cells release DAMPs and oxidized phospholipids that amplify innate immune activation, while inflammatory cytokines such as IL-6 can enhance ferroptotic susceptibility through ACSL4 induction, GPX4 suppression, and redox imbalance (He Y. et al., 2024; Kang et al., 2018; Chen et al., 2021). This bidirectional relationship argues against a single-target therapeutic model. Instead, rational intervention may require simultaneous modulation of iron availability, lipid peroxidation, cytokine signaling, and epithelial barrier injury.

Several agents with pleiotropic actions have shown promise in smoke-related or ALI models. FXR agonists such as GW4064 and natural compounds such as liquiritin attenuate pulmonary inflammation and ferroptosis while promoting tissue repair in murine models (Yang D. et al., 2024). Fluorofenidone, sea buckthorn extract, Ginkgo biloba extract, and curcumin also confer protection in COPD and smoke-related lung injury by suppressing oxidative stress, lipid peroxidation, and inflammatory signaling (Ma et al., 2021; Wu Y. et al., 2024). Among combination strategies, iron chelation plus anti-inflammatory therapy has a strong mechanistic basis. DFO reduces redox-active iron and thereby limits Fenton-driven lipid radical formation, whereas corticosteroids, IL-6 pathway inhibitors, or other anti-inflammatory agents may reduce cytokine-induced ferroptotic priming. NAC combined with iron chelation may further restore GSH-dependent antioxidant capacity and protect against oxidative lung injury (Mokra et al., 2025). Nevertheless, these combinations must be tested in SII-specific models, because excessive suppression of ROS or inflammation could impair host defense, particularly in patients at high risk for ventilator-associated pneumonia and sepsis.

### 6.3 Pharmacological constraints and delivery challenges

Despite strong mechanistic rationale, ferroptosis-targeted therapies face several pharmacological and delivery-related constraints. Many ferroptosis inhibitors have limited solubility, short half-lives, poor tissue selectivity, or off-target toxicity. Injured lungs impose additional barriers, including mucus accumulation, epithelial disruption, edema, oxidative degradation, and mucociliary clearance (Fan et al., 2025). Nanoparticle-based carriers may improve pulmonary retention and cellular targeting while reducing systemic toxicity (Yao et al., 2025a). Inhaled nanoparticles carrying GPX4 mRNA or ACSL4-directed siRNA have shown conceptual promise in preclinical ferroptosis models by limiting lipid peroxidation without fully suppressing immune function (Ni et al., 2026). Yet these systems have not been adequately validated in SII-specific experimental settings.

Safety considerations are equally important. Iron chelators may impair erythropoiesis, alter immune cell function, or aggravate anemia in burn patients. Broad antioxidants may interfere with ROS-dependent microbial killing, raising concern in patients prone to ventilator-associated pneumonia and sepsis. Radical-trapping antioxidants and lipid-modulating therapies may also interact with corticosteroids, antibiotics, anticoagulants, or ventilator-induced oxidative stress. These issues indicate that ferroptosis-targeted treatment in SII will require precise control of dose, route, timing, and patient selection rather than empirical application of anti-ferroptotic agents.

## 7 Clinical translation, biomarker-guided stratification, and future perspectives

### 7.1 Repositioning ferroptosis within the SII injury network

The current evidence supports ferroptosis as a biologically plausible and mechanistically integrated contributor to SII pathophysiology, particularly in the context of iron dysregulation after heme breakdown, lipid peroxidation induced by combustion-derived reactive species, and antioxidant depletion caused by toxic gas exposure. This interpretation must remain proportionate. SII is a multifactorial injury syndrome involving oxidative stress, innate immune activation, epithelial and endothelial damage, apoptosis, airway obstruction, vascular leakage, and fibrotic remodeling; ferroptosis does not replace these established mechanisms but intersects with them (Rehberg et al., 2009; Foncerrada et al., 2018). The available evidence, much of which derives from ALI, ARDS, COPD, hyperoxia, ischemia-reperfusion, and sepsis-associated models rather than SII-specific systems, does not justify describing ferroptosis as the dominant driver of SII. A more defensible interpretation is that ferroptosis functions as a redox-sensitive node within a broader network of regulated cell death and inflammatory amplification. Its clinical importance will likely depend on patient-specific variables, including iron burden, antioxidant reserve, inhaled toxicant profile, burn severity, infection risk, and timing of intervention (Yao et al., 2025a; Li J. et al., 2025). This calibrated view strengthens rather than weakens the translational value of ferroptosis research. SII remains a major contributor to burn-related mortality, yet treatment is still largely supportive and lacks mechanism-based pharmacological options (Enkhbaatar et al., 2016). Ferroptosis research may help address this therapeutic stagnation by linking mechanism, patient stratification, and drug repurposing. In this framework, ferroptosis is not proposed as a universal explanation for SII, but as a therapeutically relevant vulnerability that may be present in selected biological contexts.

### 7.2 Biomarker-guided patient stratification

From a translational perspective, the identification of reliable ferroptosis-associated biomarkers represents a critical step toward improved diagnosis, risk stratification, and prognostic assessment in SII and related lung disorders. Key molecular markers, including GPX4, ACSL4, ferritin, and MDA, have been consistently linked to ferroptotic activity in ALI models (Yu and Sun, 2023). Additional genes involved in iron transport and redox regulation, such as Cp, SLC39A14, and SLC7A11, correlate with neutrophil infiltration and disease severity in ALI/ARDS, underscoring their potential clinical relevance (Ma et al., 2023). Emerging metabolic markers, including elevated lactate levels associated with histone lactylation and Methyltransferase-Like 3 (METTL3) activation, further expand the ferroptosis biomarker landscape in sepsis-associated lung injury (Wu D. et al., 2024). In chronic fibrotic conditions such as IPF, ATM has also been proposed as a candidate ferroptosis-related biomarker (Yue et al., 2025).

However, these markers require validation in SII cohorts before they can guide treatment. The central challenge is specificity: many ferroptosis-associated markers also reflect generalized oxidative damage, inflammation, apoptosis, necroptosis, or tissue necrosis. Lipid peroxidation products such as MDA and 4-HNE, reduced GPX4 activity, GSH depletion, ACSL4 upregulation, ferritin alteration, and iron accumulation are frequently used as indicators of ferroptotic activity, but none is sufficiently specific in the complex injury environment of SII (Alves et al., 2025). A clinically useful biomarker system will likely need to combine iron-related indices, lipid peroxidation markers, antioxidant capacity, epithelial injury markers, and inflammatory signatures rather than relying on a single molecule. Biomarker development should also account for SII heterogeneity. Patients with predominantly thermal upper-airway injury may differ from those with prolonged particulate and toxic gas exposure. Hyperinflammatory phenotypes may exhibit greater ferroptotic activity than hypoinflammatory phenotypes because of higher cytokine burden and oxidative stress (Yao et al., 2025a). Similarly, patients with high baseline iron load, impaired GSH synthesis, or reduced GPX4 activity may be more likely to benefit from anti-ferroptotic therapy. Defining this ferroptosis-susceptible subpopulation is essential for precision intervention.

### 7.3 Imaging and dynamic monitoring of ferroptosis

Advances in detection technologies are enabling *in vivo* visualization and temporal monitoring of ferroptosis, thereby bridging mechanistic research and clinical application. Conventional approaches rely on indirect indicators such as lipid peroxidation products, mitochondrial structural damage, and pathway activation signatures (Matsuoka et al., 2025). More recently, ferroptosis-responsive fluorescent probes, two-photon luminescent ruthenium-based biothiol sensors, and artemisinin-derived Magnetic resonance imaging (MRI) probes (Art-Gd) have demonstrated the capacity to detect ferroptosis *in vivo* with high sensitivity, in some cases preceding conventional clinical readouts by 24–48 h (Zeng et al., 2023; Xu L. et al., 2024). In addition, molecular imaging strategies targeting TfR1 using superparamagnetic cubic iron oxide nanoparticles or detecting reducing reagent-resistant TfR1 dimers further strengthen the feasibility of ferroptosis-specific imaging in organ injury models (Yang W. et al., 2024). Although these technologies remain experimental, they may eventually support early detection, therapeutic-window definition, and pharmacodynamic monitoring. For SII specifically, dynamic imaging could help determine whether ferroptosis is concentrated in airway epithelium, alveolar epithelium, endothelial cells, or macrophage-rich inflammatory niches. Such spatial information would be valuable for selecting inhaled versus systemic delivery and for determining whether anti-ferroptotic treatment should be administered during the acute inflammatory phase, the early repair phase, or both.

### 7.4 Future research priorities

Despite these advances, clinical translation of ferroptosis-targeted therapies in lung injury remains in its infancy. Most evidence derives from preclinical sepsis and ARDS models, with limited first-in-human data available (Wang et al., 2025a). A proof-of-concept trial identifying DIPY as a potential adjunctive therapy for ARDS highlights the feasibility of targeting ferroptosis clinically, yet also underscores challenges related to patient heterogeneity, optimal intervention timing, and outcome selection (Chen X. et al., 2024). These factors necessitate carefully designed trials integrating biomarker-guided stratification and dynamic disease monitoring.

Several priorities should guide the next stage of investigation. First, SII-specific animal and *ex vivo* models should be established to define ferroptosis dynamics across the acute inflammatory phase and the later reparative or fibrotic phase. Second, multiparametric biomarker panels should be validated in patient-derived blood, BALF, and airway samples. Third, inhaled delivery systems, including liposomal radical-trapping antioxidants, aerosolized DFO-colloid complexes, and nanoparticle-based nucleic acid therapies, should be optimized for injured airways. Fourth, early-phase clinical trials should evaluate ferroptosis-modulating agents using biomarker-guided enrollment and dynamic monitoring. Finally, combination strategies should be assessed in the context of standard SII care rather than as isolated experimental interventions.

Collectively, continued investigation into ferroptosis is poised to reshape preventive and therapeutic strategies for SII (Table 2). Experimental evidence consistently demonstrates that inhibition of ferroptosis alleviates lung injury across diverse ALI models, particularly when integrated with existing interventions such as oxygen therapy and anti-inflammatory treatment (Wang et al., 2025a). Mechanistically, activation of endogenous protective pathways, notably Nrf2 signaling, confers resistance to ferroptosis and mitigates lung damage induced by particulate matter or sepsis (Dong et al., 2023). Ferroptosis oriented precision medicine, guided by individual iron and lipid metabolic profiles, offers a conceptual framework for personalized intervention that balances therapeutic efficacy with potential adverse effects (Kim et al., 2023). As mechanistic insights continue to accumulate, ferroptosis research is expected to advance integrated strategies encompassing prevention, detection, treatment, and prognosis of smoke-related lung injury from cellular to organismal levels.

**TABLE 2:** Therapeutic strategies and potential targets for modulating ferroptosis in SII.

Targeted step or node	Strategy class and exemplars	Expected direction on core readouts	Evidence level highlighted in review	Key translational notes
Lipid peroxidation chain reactions	Canonical ferroptosis inhibitors: Fer-1, Lip-1	Reduced ROS and reduced lipid hydroperoxides, accompanied by functional recovery signals such as improved lung function and attenuated inflammation in lung injury models	*In vitro* (bronchial epithelial cells) and *in vivo* evidence across related injury models	The pharmacokinetics and optimal therapeutic window in acute SII remain unclear
Labile iron pool or Fenton chemistry	Iron chelators: DFO, deferiprone; novel deferric amine compounds; curcumin	Reduced labile iron pool, reduced Fenton reaction driven ROS generation, and consequently reduced lipid peroxidation; decreased CXCL8 has been reported in smoke exposure contexts	Mechanistic rationale and smoke/oxidative injury context evidence; *in vitro* reversal of ferroptosis-protein alterations in hyperoxic lung injury	Side effects or limitations of existing chelators drive need for safer, more effective compounds
Oxidative stress buffering and ferroptotic signaling	Antioxidants or scavengers: NAC, vitamin E, melatonin; KEAP1/NRF2/PTGS2 signaling, ATM/p53 signaling	Increased antioxidant capacity and reduced ferroptotic signaling	Preclinical evidence in lung I/R models (NAC) and pathway-level support for melatonin/vitamin E	Strategy is broadly applicable but needs SII-specific protocol standardization and dosing window definition
Ferroptosis-inflammation feed-forward loop	FXR agonists; natural compounds	Reduced ferroptosis, accompanied by reduced ferroptosis together with decreased lung inflammation, and enhanced pulmonary regeneration	Murine model evidence	Best suited for complex SII pathobiology, but needs standardized formulations and endpoints for clinical translation
